# Preparation of 4-Flexible Amino-2-Arylethenyl-Quinoline Derivatives as Multi-Target Agents for the Treatment of Alzheimer’s Disease

**DOI:** 10.3390/molecules23123100

**Published:** 2018-11-27

**Authors:** Xiao-Qin Wang, Chu-Ping Zhao, Long-Cheng Zhong, De-Ling Zhu, De-Hao Mai, Mei-Gui Liang, Ming-Hua He

**Affiliations:** School of Pharmacy, Guangdong Medical University, Dongguan 523808, Guangdong, China; 13538678474@163.com (C.-P.Z.); 15625808258@163.com (L.-C.Z.); 15625850845@wo.cn (D.-L.Z.); 15875092028@163.com (D.-H.M.); 13071498620@163.com (M.-G.L.)

**Keywords:** Alzheimer’s disease, 2-arylethenylquinoline derivatives, multi-target, Aβ aggregation, antioxidant, neuroprotection

## Abstract

Alzheimer’s disease (AD) is a complex and multifactorial neurodegenerative disorder of aged people. The development of multitarget-directed ligands (MTDLs) to act as multifunctional agents to treat this disease is the mainstream of current research. As a continuation of our previous studies, a series of 4-flexible amino-2-arylethenylquinoline derivatives as multi-target agents was efficiently synthesized and evaluated for the treatment of AD. Among these synthesized derivatives, some compounds exhibited strong self-induced Aβ_1–42_ aggregation inhibition and antioxidant activity. The structure-activity relationship was summarized, which confirmed that the introduction of a flexible amino group featuring a *N*,*N*-dimethylaminoalkylamino moiety at the 4-position increased the Aβ_1–42_ aggregation inhibition activity, with an inhibition ratio of 95.3% at 20 μM concentration. Compound **6b_1_**, the optimal compound, was able to selectively chelate copper (II), and inhibit Cu^2+^-induced Aβ aggregation effectively. It also could disassemble the self-induced Aβ_1–42_ aggregation fibrils with a ratio of 64.3% at 20 μM concentration. Moreover, compound **6b_1_** showed low toxicity and a good neuroprotective effect against Aβ_1–42_-induced toxicity in SH-SY5Y cells. Furthermore, the step-down passive avoidance test indicated compound **6b_1_** significantly reversed scopolamine-induced memory deficit in mice. Taken together, these results suggested that compound **6b_1_** was a promising multi-target compound worthy of further study for AD.

## 1. Introduction

Alzheimer’s disease (AD) is a chronic and age-related neurodegenerative disorder characterized by memory loss and cognitive impairments [1,2]. Today, the number of dementia patients is estimated at some 46 million worldwide, and it is expected to reach 131.5 million by 2050, causing great economic and social burdens to the patients and their families [3,4,5].

The histopathologic hallmarks of AD are neurofibrillary tangles and amyloid plaques [6]. Due to its complex etiology, the pathogenesis of AD has not been completely elucidated, and multiple factors are thought to contribute to the development of AD, including deficits of acetylcholine (ACh), amyloid-β (Aβ) deposits, hyperphosphorylated tau protein, oxidative stress, dyshomeostasis of biometals and neuroinflammation [7,8,9,10,11]. 

Among these pathogenic factors of AD, Aβ production and aggregation in the brain play a crucial role in AD pathogenesis [12]. It initiates the pathogenic cascade, and induces synaptic dysfunction and causes neurotoxicity [13,14]. Aβ peptide is produced through proteolytic cleavage of the amyloid precursor protein (APP) by α, β or γ-secretase, which can aggregate into oligomers, protofibrils, and insoluble fibrils. These aggregates can result in the formation of senile plaques, and ultimately lead to the neuronal loss and dementia [15,16]. Aβ aggregates can produce neurotoxicity in many ways [17]. It can promote apoptosis, cause synaptic loss, and disrupt the cytoskeleton; It also can disrupt cellular calcium balance and loss membrane potential; Aβ aggregates can promote the generation of free radical via inflammatory response to cause oxidative stress; and it can disrupt synaptic plasticity, and inhibit hippocampal long-term potentiation (LTP); It also can bind to metal ions (especially Cu^2+^ and Zn^2+^) to form the complex of metal-Aβ, this complex can promote Aβ aggregation and reactive oxygen species (ROS) production which lead to neuron death [18,19,20,21,22,23,24,25,26]. Hence, the prevention of Aβ aggregation could serves as a rational strategy for the treatment of AD [27].

Recently, many studies show that there are high concentrations of metal ions (Cu^2+^, Zn^2+^, Fe^2+^) in AD-affected brains [28]. These metal ions can bind to Aβ peptide and promote Aβ aggregation, and the formation of Aβ plaques, and then lead to neuron death [29]. On the other hand, this interaction also leads to the formation of ROS and causes the oxidative damage of the central nervous systems (CNS) [30,31]. Thus, modulation of these metal ions in the brain has been proposed as a potential therapeutic strategy for AD treatment [32].

Oxidative stress has also been linked to early events in AD pathogenesis [33]. Oxidative damage can promote Aβ aggregation and the appearance of neurofibrillar tangles in AD [34]. Therefore, drugs with radical scavenging activities could potentially prevent AD. 

So far, there are only five drugs approved by FDA for clinical treatment of AD. They are four acetylcholinesterase inhibitors (AChEIs) such as tacrine, donepezil, galantamine, rivastigmine, and an *N*-methyl-d-aspartate (NMDA) receptor antagonist memantine. However, these drugs only improve the memory and cognitive function of AD patients, which are unable to prevent or halt progressive neurodegeneration of AD [35,36]. Therefore, it is urgent to develop more effective therapeutic drugs for curing AD.

Because of the complexity of AD and identification of many potential targets, the multi-target-directed ligand (MTDL) approach has recently attracted the attention of researchers in the pursuit of AD drugs [37,38]. Designing of MTDL widely incorporates several different pharmacophoric fragments into a single molecular, each fragment synergistically contributes to the overall activity profile of the MTDL molecule [39].

**Figure 1:**
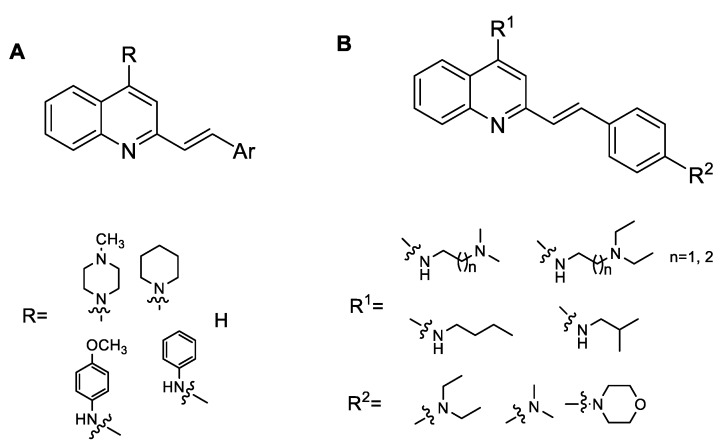
(**A**) The structure of previously described 2-arylethenylquinoline derivatives; (**B**) The structure of newly investigated compounds.

Inspired by the MTDL strategy, we previously synthesized a series of 2-arylethenylquinoline derivatives as multifunctional agents for the treatment of AD (Figure 1A) [40]. These multifunctional agents based on the 4-rigid amino or H moiety of quinoline scaffold. However, these compounds had unsatisfactory inhibitory activities of Aβ aggregation. On the other hand, our docking study of the compound and Aβ showed that there is a lot of room at the 4-position of the quinoline ring, where large substituent can be introduced to enhance the interaction of the compound with Aβ [41]. In order to improve Aβ aggregation inhibition properties of the compound, in this work, based on the structure-activity relationships (SAR) of our previous work, we introduced the flexible amino substituent at the 4-position of the quinoline ring, and synthesized a series of 4-flexible amino-2-arylethenylquinoline derivatives (Figure 1B), we also optimized the synthesis method of the target compounds, which improved the yield and shortened the reaction time; and then evaluated their biological activities, including inhibition of Aβ aggregation, antioxidative activity, metal chelating property, neuroprotection and cytotoxicity. The SAR of synthesized compounds was discussed. Furthermore, the optimal compound **6b_1_** was tested in AD mice model for the behavioral evaluations in vivo.

## 2. Results and Discussion

### 2.1. Chemistry

**Scheme 1:**
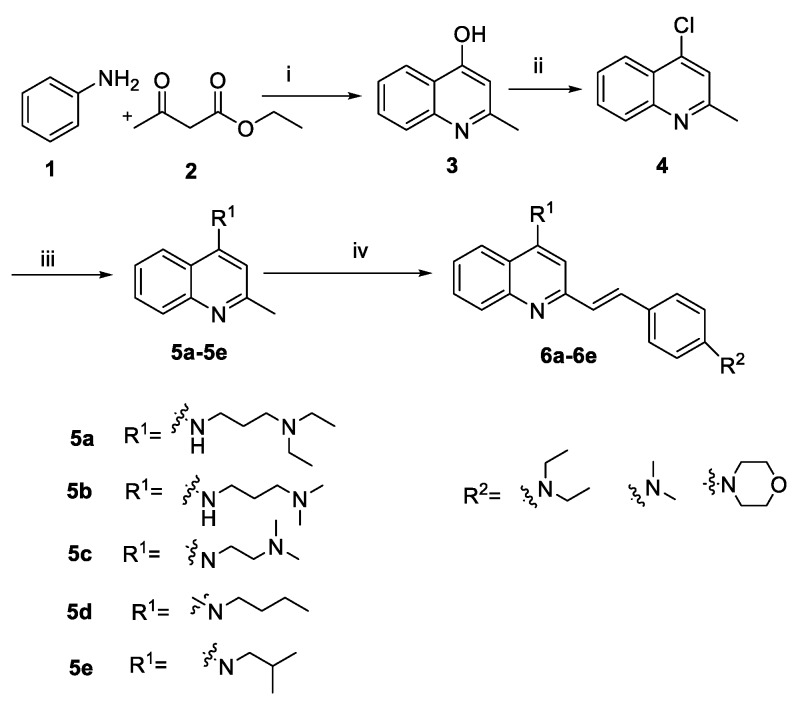
Synthetic route of 2-arylethenylquinoline derivatives **6a**–**6e**. *Reagents and conditions*: (i) PPA, 130 °C; (ii) POCl_3_, 120 °C; (iii) flexible amine, TsOH, MW, 120 °C, 1 h; (iv) aromatic aldehyde, TMSCl, 150 °C, DMF.

The synthesis of 4-substituted-2-aryethenylquinoline derivatives was previously reported by our group [40]. Herein differently substituted flexible amino-containing groups were introduced at the 4-postion of the quinoline ring, and the synthetic method of the target compounds **6a**–**6e** was optimized as illustrated in Scheme 1. 

The intermediate **4** was obtained following the procedure of our previous work [40]. The reaction of intermediate **4** with different flexible amines (for example, 3-diethylaminopopylamine, 3-dimethylaminopopylamine, *N*,*N*-dimethylethylenediamine, n-butyl-amine, or isobutylamine) catalyzed by p-toluenesulfonic acid (TsOH) at 120 °C for 1 h under microwave-assisted conditions gave the compounds **5a**–**5e**, offered several advantages such as higher yields, shorter reaction times, lower costs, more convenience, and higher efficiency compared to our previous synthetic method [42]. Finally, the target compounds **6a**–**6e** (**6a_1_**–**6a_4_**, **6b_1_**–**6b_3_**, **6c_1_**–**6c_2_**, **6d_1_**–**6d_2_**, and **6e_1_**–**6e_2_**) were obtained by the reaction of compounds **5a**–**5e** with different substituent aromatic aldehydes in the presence of trimethylchlorosilane (TMSCl) at 150 °C for 24 h, The yields of compounds **6a**–**6e** were in range of 71%–85%. This synthetic method shortened the reaction time, and improved the product yield. The structures of the target compounds were validated by using ^1^H-NMR, ^13^C-NMR, and HRMS, and their purities were determined to be above 95% by using HPLC.

### 2.2. Inhibition of Aβ_1–42_ Self-Induced Aggregation

The formation and aggregation of Aβ_1–42_ in the brain leads to neurotoxicity in AD. In order to evaluate the activity of our new synthetic compounds to inhibit Aβ_1–42_ self-induced aggregation, the thioflavin (ThT) fluorescence assay was performed [40,43]. Resveratrol and curcumin were used as reference compounds. The results are shown in Table 1. 

It is seen that all of the compounds exhibited strong inhibitory activity (>77% at 20 μM), which is higher than those of curcumin (49.3% at 20 μM) and resveratrol (77.2% at 20 μM). Compound **6b_1_**, **6b_2_**, and **6a_1_** showed the most potent inhibitory activities, with respective inhibition ratio of 95.3%, 92.1% and 90.2%. In order to further investigate their dose-dependent inhibition of Aβ_1–42_ self-induced aggregation, the IC_50_ values of compound **6b_1_**, **6b_2_**, and **6a_1_** were determined. The result is shown in Table 2, they exhibited better inhibition than resveratrol (IC_50_ = 11.8 μM), with the IC_50_ values of compound **6b_1_**, **6b_2_**, and **6a_1_** were 4.5 μM, 6.1 μM, and 7.8 μM, respectively. The structure-activity relationship was also explored. The influence of the substituent in the benzene ring of arylethenyl part on Aβ aggregation inhibition has been studied in our previous work [40]. Previous studies showed that the substituent group with 4-dimethylamino or 4-diethylamino on the benzene ring is favorable for the inhibitory activity. Here, we mainly investigated the effect of substituent at the 4-postion of quinoline ring on Aβ aggregation inhibition.

Firstly, the diamino substitution group at the 4-postion of quinoline ring obviously increased the inhibitory activity (the Aβ aggregation inhibitory values of the series of **a**, **b**, and **c** compared to the series of **d**, **e**, respectively). Suggesting that amino substituents at 4-postion of quinoline ring played an important role in the inhibition of Aβ aggregation, which is consistent with our previous experimental results. Secondly, the type of diamino substituents had a great effect on the inhibitory activity of the compounds, the substituent group featured with *N*,*N*-dimethylaminoalkylamino at 4-postion of quinoline scaffold gave better inhibitory activity than that featured with *N*,*N*-diethylaminoalkylamino (the Aβ_1–42_ aggregation inhibitory values of the series of **b** compared to the series of **a**, respectively). This may be because *N*,*N*-diethylaminoalkylamino gives larger space resistance.

In addition, some target compounds with different linker length between two *N* atoms at 4-postion of quinoline scaffold were synthesized and evaluated. It was found that compound **6a** and **6b** with three-carbon atom linker exhibited better Aβ aggregation inhibition than compound **6c** with two-carbon atom linker.

Finally, we also compared the inhibitory activity of the new 4-flexible amino-2-arylethenylquinoline derivatives with compound **4b_1_** and **4b_2_** with 4-rigid amino substituents in the quinoline scaffold which were previously described for the best Aβ aggregation inhibition [40]. It was found that flexible amino at 4-postion of quinoline scaffold contributed to the increased activity.

### 2.3. Antioxidant Activity In Vitro

Oxidative stress is another crucial event in AD pathogenesis. In order to determine antioxidant activities of our synthetic compounds, the oxygen radical absorbance capacity method with fluorescein (ORAC-FL) assay was performed [44,45]. with Trolox (6-hydroxy-2,5,7,8-tetramethylchroman-2-carboxylic acid) as the standard, and their antioxidant activity was expressed as Trolox equivalents. The data is shown in Table 1. Our data suggested that most of the compounds demonstrated good antioxidant activities. Compounds **6a_1_**, **6b_1_**, and **6a_2_** exhibited the most potent antioxidative action, with ORAC-FL values of 6.85, 6.54 and 6.28 at a concentration of 5 μM, respectively, which was better than that of our previously synthetic compounds **4b_1_** and **4b_2_**. In addition, comparing the antioxidative activity of series **6d** and **6e**, the series of compounds **6a**, **6b** and **6c** with a diamino substitutent at the 4-postion of the quinoline ring had better antioxidative activities. This might be because that amino substituent is crucial for the radical scavenging ability.

### 2.4. Antioxidant Activity in SH-SY5Y Cells

**Figure 2:**
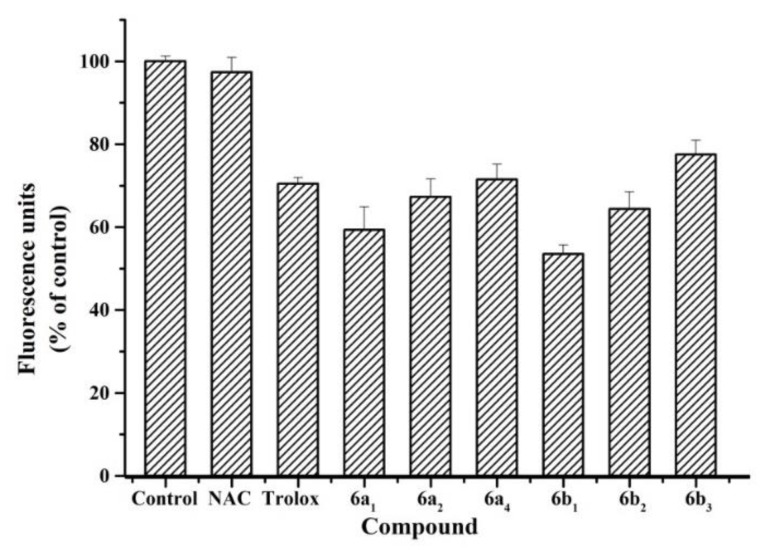
ROS generation in SH-SY5Y cells incubated without or with compounds measured using DCFH-DA. The results are expressed as the percentage of control cells (without compounds).

To further investigate their antioxidant activity in SH-SY5Y cells, cellular ROS detection assay based on dichlorofluorescein diacetate (DCFH-DA) was performed [46]. with Trolox and *N*-acetyl-l-cysteine (NAC) as reference compounds. Compounds that showed higher antioxidant activity in vitro and self-induced Aβ_1–42_ aggregation inhibition were selected, and the concentration of the tested compounds was 2.5 μM which had no effect on the cell viability. As shown in Figure 2, the tested compounds and Trolox could decrease the intensity of fluorescence differently, but NAC didn’t remove ROS generation in SH-SY5Y cells treated with t-BuOOH. Compound **6b_1_** and **6a_1_** displayed more antioxidant activity than Trolox, and compound **6a_4_** and **6b_3_** exhibited a little weaker antioxidant activity than Trolox. These results indicated that the mechanism of these compounds abolish ROS generation might be similar to that of Trolox, which is consistent with our previous experiment findings.

Considering that compound **6b_1_** showed more active than other compounds in inhibiting self-induced Aβ_1–42_ aggregation and antioxidant activity, compound **6b_1_** was selected for further study.

### 2.5. Metal Binding Properties of Compound **6b_1_**

**Figure 3:**
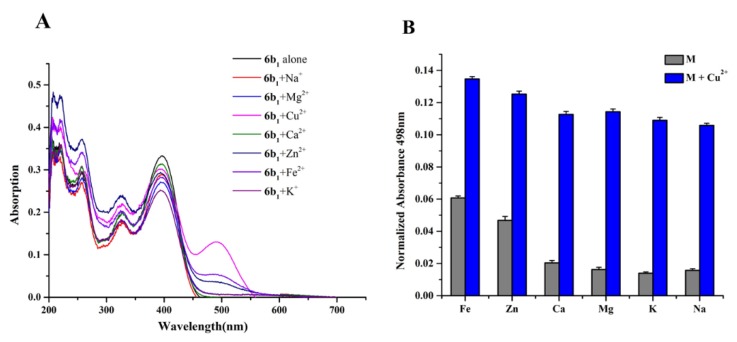
(**A**) UV-vis absorption spectra of compound **6b_1_** (10 μM) alone or in the presence of 20 μΜ NaCl, KCl, MgCl_2_, CaCl_2_, CuSO_4_, ZnSO_4_, and FeSO_4_. (**B**) Metal selectivity for compound **6b_1_**. Relative changes in optical response (absorbance intensity) when the compound interacted with metal ions. Gray bars represent normalized affinity for metal ions M (M = Zn^2+^, Fe^2+^, Ca^2+^, Mg^2+^, K^+^ or Na^+^), while blue bars indicate affinity for Cu^2+^ in presence of M.

The chelation ability of compound **6b_1_** toward biometals such as Na^+^, K^+^, Cu^2+^, Fe^2+^, Mg^2+^, Ca^2+^, and Zn^2+^ was studied by UV-vis spectrometry [47,48]. When Cu^2+^, Fe^2+^, and Zn^2+^ was added, new optical bands were observed at about 498 nm, and the peak at about 393.5 nm which was observed on UV spectrum of compound **6b_1_** in ethanol alone decreased, indicative of the interaction between Cu^2+^, Fe^2+^, Zn^2+^ and compound **6b_1_** to form the complex **6b_1_**-metal (Ⅱ). But there was little change on the UV spectrum upon the addition of Na^+^, K^+^, Mg^2+^, and Ca^2+^ (shown in Figure 3A), which indicated that compound **6b_1_** may not bind to these metal ions. Interestingly, after CuSO_4_ was added, the absorption at 498 nm increased obviously, this may be because compound **6b_1_** complexed with Cu^2+^ strongly. Its ability to selectively chelate Cu^2+^ versus other relevant metal ions was further investigated by UV-vis spectrometry. Solution of compound **6b_1_** was prepared and treated with the metal ion (Zn^2+^, Fe^2+^, Ca^2+^, Mg^2+^, Na^+^ and K^+^), after 10 min incubation, the optical response was observed, followed by addition of Cu^2+^ and consequent analysis of further spectral changes. The result is shown in Figure 3B. It can be seen that compound **6b_1_** had good selectivity of metal chelation for Cu^2+^. 

**Figure 4:**
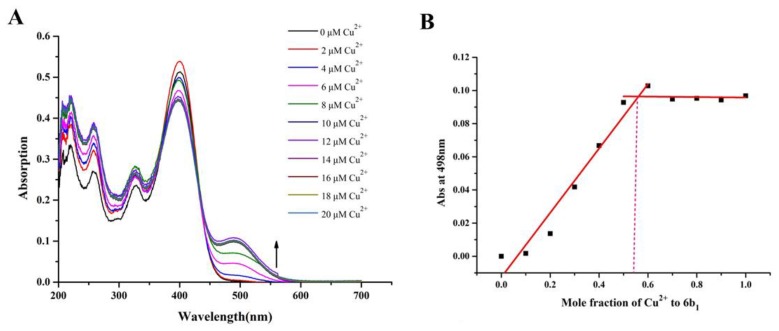
(**A**) UV-vis absorption spectra of compound **6b_1_** (20 μM) in ethanol after addition of ascending amount of CuSO_4_ (0–20 μΜ). (**B**) Determination of the stoichiometry of complex **6b_1_**-Cu^2+^ by molar ration method.

In order to further determine the stoichiometry of compound **6b_1_** for Cu^2+^ binding, the UV spectra was performed by titrations of compound **6b_1_** upon stepwise additions of a Cu^2+^ solution. According to Figure 4A, the absorbance at 498 nm firstly increased with ascending amount of CuSO_4_, and then tended to be stable, indicating that the chelation reached saturation point. The molar ratio was calculated (Figure 4B), two straight lines were drawn with the intersection point at a mole fraction of 0.5, revealing a 2:1 stoichiometry for complex **6b_1_**-Cu^2+^ (compound/Cu^2+^; binding maybe through *N* atoms of 4-flexible amine group, the chelating motif *NH*-*N* is proposed. See Scheme S1).

### 2.6. Inhibition of Cu^2+^-Induced Aβ_1–42_ Aggregation

**Figure 5:**
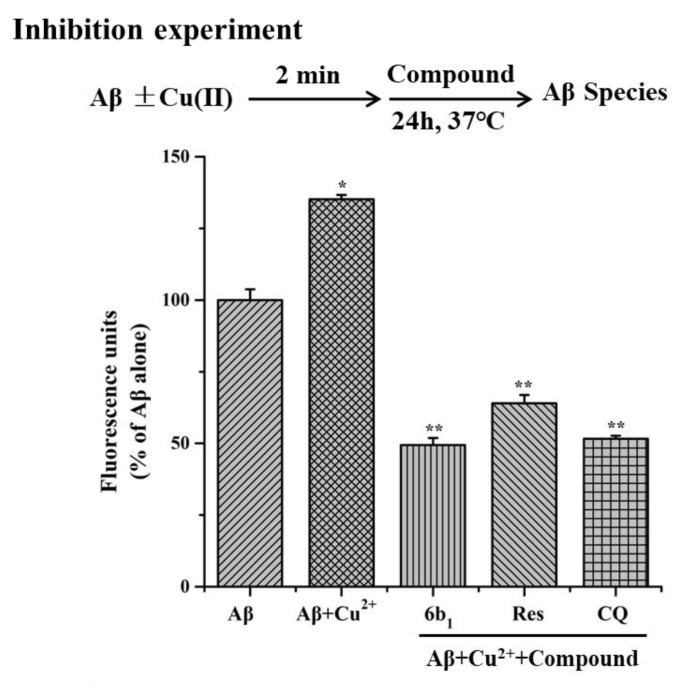
Inhibition of Cu^2+^-induced Aβ_1–42_ aggregation by compound **6b_1_** comparing with those of resveratrol (Res) and clioquinol (CQ) ([Aβ] = 20 μM, [Cu^2+^] = 20 μM, [compound] = 40 μM). Values are reported as the mean ± SD of three independent experiments. *p < 0.05 vs. Aβ, **p < 0.01 vs. Aβ treated with Cu^2+^.

To investigate the ability of compound **6b_1_** to inhibit Cu^2+^-induced Aβ_1–42_ aggregation, the ThT-binding assay was performed [49]. Resveratrol and clioquinol (CQ) were used as reference compounds. As shown in Figure 5, the fluorescence of Aβ treated with Cu^2+^ is 135.2% that of Aβ alone, which indicated that Cu^2+^ accelerated Aβ aggregation. But treated with the compounds, the fluorescence of Aβ treated with Cu^2+^ decreased differently. Compound **6b_1_** displayed 85.8% inhibition of Cu^2+^-induced Aβ aggregation, which was equal to CQ (83.6% inhibition of Cu^2+^-induced Aβ aggregation); Resveratrol displayed weaker inhibition (71.2% inhibition of Cu^2+^-induced Aβ aggregation). These results suggested that compound **6b_1_** could inhibit Cu^2+^-induced Aβ aggregation effectively.

### 2.7. Cytotoxic Effect on SH-SY5Y

The cytotoxicity of our synthesized compounds was examined in human neuroblastoma SH-SY5Y cells. The cell viability was determined by using methyl thiazolyl tetrazolium (MTT) colorimetry, the cells were treated with different concentrations of compounds (0–100 μM) [50]. The result is shown in the Supplementary Materials (Table S1). Our data indicated that all the compounds have their IC_50_ values above 100 μM, which implied that all the compounds have low cytotoxicity. In addition, compounds **6b_1_**, **6b_2_**, and **6a_1_** with higher self-induced Aβ_1–42_ aggregation inhibition exhibited lowest cytotoxicity with their IC_50_ values of 253.7 μM, 228.1 μM and 189.2 μM, respectively.

### 2.8. Effect of Compound 6b_1_ on Abundance of Aβ_1–42_ Fibrils

**Figure 6:**
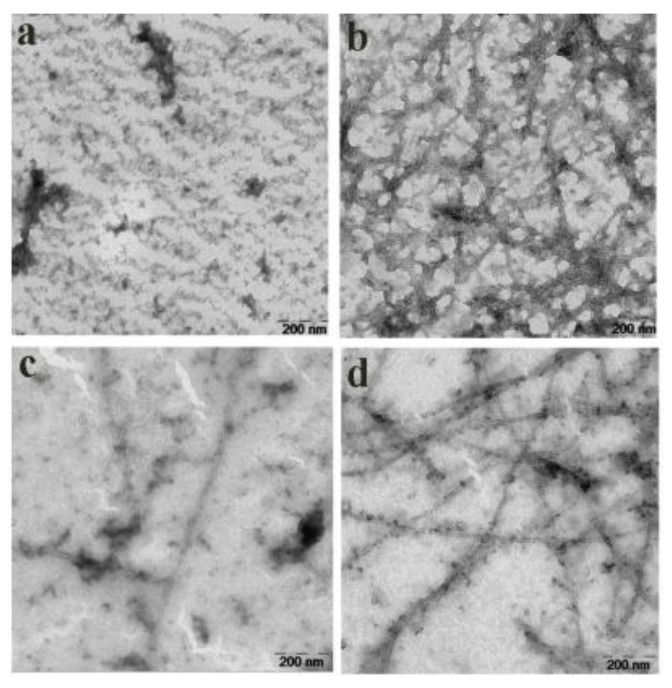
TEM image analysis of Aβ_1–42_ aggregation in the presence and absence of the compound. (**a**) Aβ_1–42_ (20 μM), 0 h. (**b**) Aβ_1–42_ (20 μM) was incubated at 37 °C for 24 h. (**c**) Aβ_1–42_ (20 μM) and **6b_1_** (20 μM) were incubated at 37 °C for 24 h. (**d**) Aβ_1–42_ (20 μM) and resveratrol (20 μM) were incubated at 37 °C for 24 h.

To further complement the ThT binding assay, A transmission electron microscopy (TEM) assay was employed to monitor and clarify the effect of compound **6b_1_** on Aβ_1−42_ aggregation [51,52]. As shown in Figure 6, after 24 h incubation at 37 °C, the sample of Aβ_1−42_ alone had aggregated into many amyloid fibrils (Figure 6b), while a few thick fibrils were observed for the sample of Aβ_1–42_ in the presence of resveratrol (Figure 6d). Compared to resveratrol, only fewer thin fibrils and small bulk aggregates were observed in the sample of Aβ_1−42_ in the presence of **6b_1_** (Figure 6c). The TEM result was well consistent with the result of ThT, which strongly proved that compound **6b_1_** had better inhibition against Aβ_1−42_ fibrils formation than resveratrol.

### 2.9. Disaggregation of Self-Induced Aβ_1–42_ Aggregation Fibrils by 6b_1_

**Figure 7:**
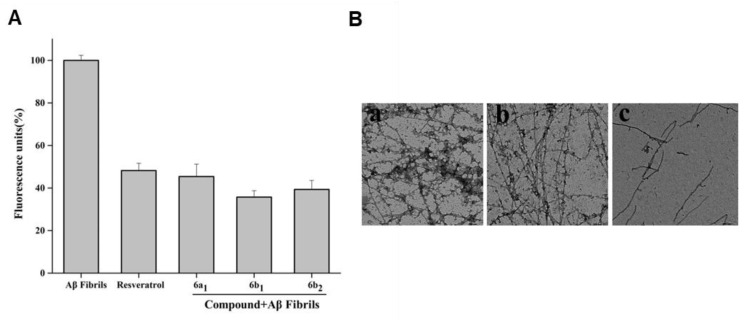
(**A**) Results of the ThT binding assay for Aβ_1–42_ without and with test compound. (**B**) TEM images for Aβ_1–42_ disaggregation. (**a**) Aβ_1–42_ (20 μM) was incubated at 37 °C for 24 h in phosphate buffer. (**b**) Aβ_1–42_ (20 μM) was incubated with resveratrol (20 μM) at 37 °C for 24 h. (**c**) Aβ_1–42_ (20 μM) was incubated with **6b_1_** (20 μM) at 37 °C for 24 h.

The ability of compound **6b_1_** to disaggregate self-induced Aβ_1–42_ aggregation fibrils was also investigated [52]. Aβ_1–42_ fibrils were prepared by incubating fresh Aβ_1–42_ for 24 h at 37 °C, and the test compound was added to the sample and incubated for another 24 h at 37 °C. Then the sample was analyzed by ThT binding assay and TEM assay. The ThT binding assay exhibited compound **6b_1_** disaggregated Aβ_1–42_ fibrils with ratio of 64.3% at 20 μM concentration, and resveratrol showed weaker activity with ratio of 51.8%, as shown in Figure 7A. Our TEM results exhibited compound **6b_1_** incubated with Aβ_1–42_ fibrils could disaggregate more Aβ_1–42_ fibrils than resveratrol (Figure 7B), which further supported the result of the ThT binding assay.

### 2.10. In vitro Protective Effect of Compound 6b_1_ against Aβ_1–42_ Induced Toxicity in SH-SY5Y Human Neuroblastoma Cells

**Figure 8:**
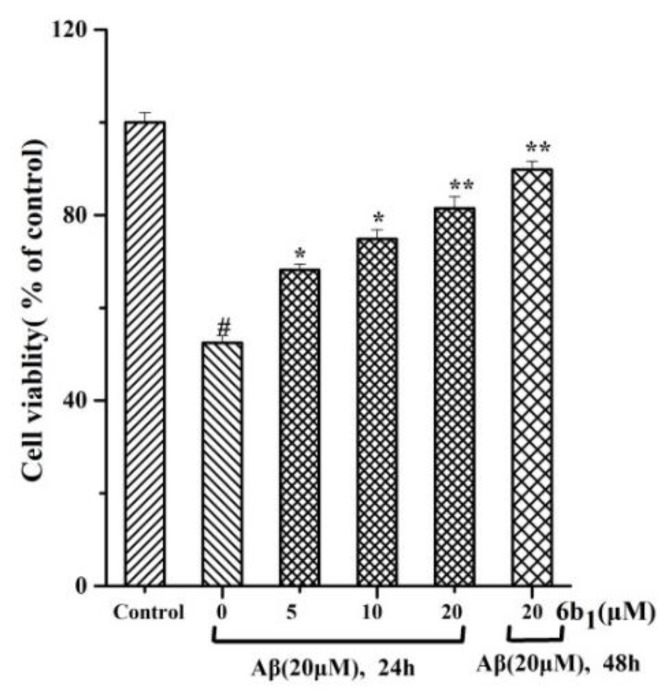
Cytoprotective effect of **6b_1_** on SH-SY5Y human neuroblastoma cells. Viability was measured by MTT assay after 24 and 48 h of incubation at 37 °C with Aβ_1–42_ (20 μM) in the absence and presence of **6b_1_**. The percentage of MTT reduction is relative to control cells in medium. Values are expressed as the mean ± SEM (*n* = 6), ^#^ *p* < 0.05 vs. control; * *p* < 0.05 and ** *p* < 0.01 vs. Aβ.

The cytoprotective effect of compound **6b_1_** against Aβ_1–42_ damage in SH-SY5Y human neuroblastoma cell lines was determined by MTT assay [53,54]. Our cytotoxicity study had indicated that compound **6b_1_** does not affect cell viability at the concentration of 20 μM. However, incubation of Aβ_1–42_ (20 μM) with SH-SY5Y cells for 24 h resulted in a 47.5% reduction viability, compared with the control group (untreated group). To investigate the protective effect of compound **6b_1_** on Aβ_1–42_ induced cell toxicity, SH-SY5Y cells exposed to Aβ_1–42_ (20 μM) were incubated with different concentrations of compound **6b_1_** (5, 10, and 20 μM) for 24 h, and the cell viability was tested. As shown in Figure 8, treatment with compound **6b_1_** increased the cell viability by preventing Aβ_1–42_-induced cytotoxicity in a concentration-dependent manner. Furthermore, when the SH-SY5Y cells were co-incubated with compound **6b_1_** and Aβ_1–42_ for 48 h, the cell viability increased to 89.8%. All these results indicated that compound **6b_1_** exhibited a neuroprotective role against Aβ_1–42_-induced cell toxicity.

### 2.11. Step-Down Type Passive Avoidance Test

**Figure 9:**
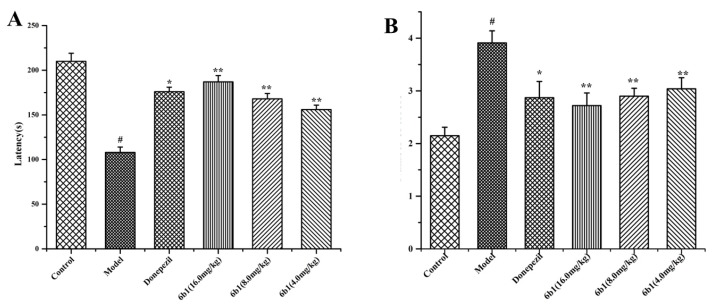
Effects of compound **6b_1_** on the (**A**) latency (s) and (**B**) number of errors in the step-down test by the scopolamine-induced cognitive impairment. The data shown are mean ± SEM (*n* = 8). ^#^ *p* < 0.01 vs. control group, * *p* < 0.05, ** *p* < 0.01 vs. model group.

In order to determine whether compound **6b_1_** could improve the memory impairment in scopolamine-induced mice, the step-down passive avoidance test was performed [55,56], donepezil was used as positive control. The doses of the tested compounds were not toxic to the mice. As shown in Figure 9, the model group treated with scopolamine alone exhibited much shorter latency and more number of errors than the control group. While treatment with donepezil group (5 mg/kg) showed longer latency time (176 s) and less number of errors (2.87) than the model group with scopolamine, which indicated donepezil significantly reversed the cognitive impairment induced by scopolamine. Subsequently, treatment with compound **6b_1_** (4.0, 8.0 and 16.0 mg/kg) increased the latency and reduced the number of errors in a dose-dependent manner. For the low dose group of compound **6b_1_** (4.0 mg/kg), it didn’t exhibit significant improvement of the memory impairment compared with the model group. However, the high dose group (16.0 mg/kg) presented the longest latency time (182 s) and least number of errors (2.72), which was better than donepezil group. In general, these results demonstrated that compound **6b_1_** could improve cognitive deficit induced by scopolamine.

## 3. Materials and Methods 

### 3.1. General Information 

All commercial reagents and solvents were purchased from commercial suppliers and used without further purification. ^1^H- and ^13^C-NMR spectra were recorded using TMS as the internal standard in CDCl_3_ or DMSO-*d_6_* with a Bruker BioSpin GmbH spectrometer (Billerica, MA, USA) at 400 MHz and 101 MHz, respectively. High resolution mass spectra (HRMS) were recorded using Shimadzu LCMS-IT-TOF spectrometer (Shimadzu, Kyoto, Japan). Melting points (mp) were obtained using a SRS-OptiMelt automated melting point instrument (Sunnyvale, CA, USA) without correction. The purities of synthesized compounds were confirmed to be higher than 95% by analytical HPLC performed with a dual pump Shimadzu LC-20AB (Shimadzu) system equipped with a Ultimate XB-C18 column and eluted with methanol-water (60:40–70:30) containing 0.1% TFA at a flow rate of 0.3 mL/min. Flash column chromatography was performed with silica gel (200–300 mesh) purchased from Qingdao Haiyang Chemical Co. Ltd. (Qingdao, China). All the reactions were monitored by thin layer chromatography using silica gel.

### 3.2. Chemistry

#### 3.2.1. Synthesis of Intermediates

The intermediates **3**, **4** were prepared following the procedure shown in Scheme 1 using our previous reported method [40]. Compounds **5a**–**5e** were synthesized under microwave-assisted organic synthesis (MAOS) conditions with high yield by our recent reported method [42]. 

#### 3.2.2. General Procedure A for the Synthesis of Compounds **6a**–**6e**

A mixture of compound **5a**–**5e** (1.0 mmol), different aromatic aldehyde (1.05 mmol), and trimethylchlorosilane (2 mL) in DMF (5 mL) was heated at 150 °C for 24 h. The reaction mixture was allowed to cool to room temperature, and poured into ice water (40 mL), and then aqueous NaOH was added to neutralize the solution to generate precipitate. The precipitate was filtered and washed with water, and the crude product was purified by using flash column chromatography with EtOAc/methanol (50:1) or CH_2_Cl_2_/petroleum ether (3:1) elution to afford the desired products. 

*(E)-N^1^-(2-(4-(Diethylamino)styryl)quinolin-4-yl)-N^3^,N^3^-diethylpropane-1,3-diamine* (**6a_1_**)

Compound **5a** was reacted with 4-diethylaminobenzaldehyde following the general procedure A to afford product **6a_1_** as an orange solid in 72% yield. mp. 182.3–184.0 °C. ^1^H-NMR (400 MHz, CDCl_3_): δ 8.00 (s, 1H), 7.70 (d, *J* = 7.5 Hz, 1H), 7.60–7.53 (m, 2H), 7.51 (d, *J* = 8.8 Hz, 2H), 7.31 (t, *J* = 7.6 Hz, 1H), 7.12 (d, *J* = 8.8 Hz, 1H), 6.67 (d, *J* = 8.9 Hz, 2H), 6.57 (s, 1H), 3.49 (dd, *J* = 10.0, 5.5 Hz, 2H), 3.39 (q, *J* = 7.1 Hz, 4H), 2.75–2.69 (m, 2H), 2.66 (q, *J* = 7.1 Hz, 4H), 1.99–1.93 (m, 2H), 1.19 (t, *J* = 7.1 Hz, 6H), 1.11 (t, *J* = 7.1 Hz, 6H). ^13^C-NMR (101 MHz, CDCl_3_): δ 157.40, 151.21, 150.34, 148.06, 134.13, 130.28, 129.34, 128.78, 125.46, 125.11, 123.89, 122.87, 118.05, 112.58, 95.78, 53.66, 47.17, 44.38, 41.26, 23.88, 12.68, 12.16. Purity: 99.2% by HPLC. HRMS (ESI): Cacld for [M + H]^+^ (C_28_H_39_N_4_) requires *m/z* 431.3169, found 431.3188.

*(E)-N^1^-(2-(4-(Dimethylamino)styryl)quinolin-4-yl)-N^3^,N^3^-diethylpropane-1,3-diamine* (**6a_2_**)

Compound **5a** was reacted with 4-dimethylaminobenzaldehyde following the general procedure A to afford product **6a_2_** as an orange-red solid in 74% yield. mp. 168.5–170.1 °C. ^1^H-NMR (400 MHz, CDCl_3_): δ 7.96 (d, *J* = 8.3 Hz, 1H), 7.70 (d, *J* = 3.4 Hz, 1H), 7.59–7.57 (m, 1H), 7.55–7.50 (m, 3H), 7.31 (t, *J* = 8.8 Hz, 1H), 7.12 (d, *J* = 16.2 Hz, 1H), 6.72 (d, *J* = 8.8 Hz, 2H), 6.57 (s, 1H), 3.48 (dd, *J* = 10.1, 5.6 Hz, 2H), 3.00 (s, 6H), 2.69 (t, *J* = 3.4 Hz, 2H), 2.65 (q, *J* = 4.4 Hz, 4H), 1.99–1.91 (m, 2H), 1.11 (t, *J* = 7.1 Hz, 6H). ^13^C-NMR (101 MHz, CDCl_3_): δ 157.30, 150.69, 150.53, 148.58, 133.37, 130.62, 128.95, 128.40, 125.69, 125.25, 123.40, 120.29, 118.62, 112.29, 95.49, 53.45, 47.09, 44.55, 40.34, 24.72, 11.58. Purity: 99.7% by HPLC. HRMS (ESI): Cacld for [M + H]^+^ (C_26_H_35_N_4_) requires *m/z* 403.2856, found 403.2866.

*(E)-N^1^-(2-(2-(1H-Indol-3-yl)vinyl)quinolin-4-yl)-N^3^,N^3^-diethylpropane-1,3-diamine* (**6a_3_**)

Compound **5a** was reacted with indole-3-formaldehyde following the general procedure A to afford product **6a_3_** as a pale yellow solid in 74% yield. mp. 194.3–195.1 °C. ^1^H-NMR (400 MHz, CDCl_3_): δ 8.98 (s, 1H), 8.08 (d, *J* = 7.4 Hz, 1H), 8.02 (d, *J* = 8.4 Hz, 1H), 7.88 (d, *J* = 16.3 Hz, 1H), 7.71 (d, *J* = 8.3 Hz, 1H), 7.58 (t, *J* = 7.6 Hz, 1H), 7.47 (s, 1H), 7.39 (d, *J* = 7.6 Hz, 1H), 7.33–7.26 (m, 2H), 7.22–7.15 (m, 2H), 6.53 (s, 1H), 3.48 (t, *J* = 3.3 Hz, 2H), 2.70 (t, *J* = 8.0 Hz,2H), 2.67 (q, *J* = 14.2 Hz, 4H), 1.98–1.91 (m, 2H), 1.11 (t, *J* = 7.1 Hz, 6H). ^13^C-NMR (101 MHz, CDCl_3_): δ 157.37, 150.99, 147.79, 137.22, 129.28, 128.25, 127.47, 126.40, 125.67, 124.85, 123.45, 122.49, 120.47, 120.42, 120.38, 118.42, 114.88, 111.78, 95.36, 53.40, 47.04, 44.57, 24.60, 11.51. Purity: 97.2% by HPLC. HRMS (ESI): Cacld for [M + H]^+^ (C_26_H_31_N_4_) requires *m/z* 399.2543, found 399.2552.

*(E)-N^1^,N^1^-Diethyl-N^3^-(2-(4-morpholinostyryl)quinolin-4-yl)propane-1,3-diamine* (**6a_4_**)

Compound **5a** was reacted with 4-morpholinyl-benzaldehyde following the general procedure A to afford product **6a_4_** as an orange solid in 76% yield. mp. 172.5–174.3 °C. ^1^H-NMR (400 MHz, CDCl_3_): δ 7.97 (d, *J* = 8.4 Hz, 1H), 7.80 (s, 1H), 7.73 (d, *J* = 7.6 Hz, 1H), 7.61–7.52 (m, 4H), 7.32 (t, *J* = 11.1 Hz, 1H), 7.17 (d, *J* = 16.2 Hz, 1H), 6.90 (d, *J* = 8.8 Hz, 2H), 6.56 (s, 1H), 3.89–3.83 (m, 4H), 3.46 (t, *J* = 5.5 Hz, 2H), 3.24–3.17 (m, 4H), 2.71–2.63 (m, 6H), 1.99–1.90 (m, 2H), 1.10 (t, *J* = 7.1 Hz, 6H). ^13^C-NMR (101 MHz, CDCl_3_): δ 158.14, 151.23, 149.68, 143.61, 133.40, 129.53, 129.07, 128.34, 125.56, 124.48, 123.70, 120.75, 113.36, 106.58, 96.77, 66.80, 53.67, 53.10, 47.93, 44.21, 24.54, 11.99. Purity: 98.6% by HPLC. HRMS (ESI): Cacld for [M + H]^+^ (C_28_H_37_N_4_O) requires *m/z* 445.2962, found 445.2979.

*(E)-N^1^-(2-(4-(Diethylamino)styryl)quinolin-4-yl)-N^3^,N^3^-dimethylpropane-1,3-diamine* (**6b_1_**)

Compound **5b** was reacted with 4-diethylaminobenzaldehyde following the general procedure A to afford product **6b_1_** as an orange-red solid in 73% yield. mp 153.9–155.2 °C. ^1^H-NMR (400 MHz, CDCl_3_): δ 8.36 (s, 1H), 7.71 (d, *J* = 16.1 Hz, 1H), 7.64–7.57 (m, 2H), 7.54 (d, *J* = 8.9 Hz, 2H), 7.34 (t, *J* = 4.0 Hz, 1H), 7.25 (t, *J* = 16.1Hz, 1H), 6.65 (d, *J* = 8.9 Hz, 2H), 6.49 (s, 1H), 3.57–3.50 (m, 2H), 3.40 (q, *J* = 8.0 Hz, 4H), 2.64 (t, *J* = 8.0 Hz, 2H), 2.40 (s, 6H), 2.34 (s, 1H), 2.01–1.96 (m, 2H), 1.20 (t, *J* = 8.0 Hz, 6H). ^13^C-NMR (101 MHz, CDCl_3_): δ 154.47, 152.29, 148.67, 143.43, 137.10, 130.50, 129.51, 124.81, 124.56, 123.02, 120.78, 119.07, 117.52, 111.40, 93.95, 58.72, 45.37, 44.41, 43.66, 24.68, 12.65. Purity: 97.0% by HPLC. HRMS (ESI): Cacld for [M + H]^+^ (C_26_H_35_N_4_) requires *m/z* 403.2856, found 403.2880.

*(E)-N^1^-(2-(4-(Dimethylamino)styryl)quinolin-4-yl)-N^3^,N^3^-dimethylpropane-1,3-diamine* (**6b_2_**)

Compound **5b** was reacted with 4-dimethylaminobenzaldehyde following the general procedure A to afford product **6b_2_** as an orange solid in 75% yield. mp 151.2–153.3 °C. ^1^H-NMR (400 MHz, CDCl_3_): δ 7.94 (d, *J* = 8.3 Hz, 1H), 7.56–7.48 (m, 3H), 7.45(d, *J* = 8.0 Hz, 2H), 7.24 (t, *J* = 4.4 Hz, 1H), 7.05 (d, *J* = 16.2 Hz, 1H), 6.63 (d, *J* = 8.8 Hz, 2H), 6.49 (s, 1H), 3.51–3.30 (m, 2H), 2.92 (s, 6H), 2.49 (t, *J* = 8.0 Hz, 2H), 2.28 (s, 6H), 2.05–1.68 (m, 2H). ^13^C-NMR (101 MHz, CDCl_3_): δ 157.22, 150.65, 149.62, 142.17, 134.07, 129.37, 128.50, 125.08, 124.28, 123.67, 121.42, 119.52, 117.95, 112.23, 94.40, 57.88, 44.71, 43.73, 41.54, 26.19. Purity: 97.0% by HPLC. HRMS (ESI): Cacld for [M + H]^+^ (C_24_H_31_N_4_) requires *m/z* 375.2543, found 375.2562.

*(E)-N^1^-(2-(2-(1H-Indol-3-yl)vinyl)quinolin-4-yl)-N^3^,N^3^-dimethylpropane-1,3-diamine* (**6b_3_**)

Compound **5b** was reacted with indole-3-formaldehyde following the general procedure A to afford product **6b_3_** as a pale yellow solid in 77% yield. mp. 168.2–169.5 °C. ^1^H-NMR (400 MHz, CDCl_3_): δ 9.30 (s, 1H), 8.03 (t, *J* = 8.4 Hz, 2H), 7.85 (d, *J* = 16.3 Hz, 1H), 7.71 (s, 1H), 7.62–7.55 (m, 2H), 7.44 (s, 1H), 7.38 (d, *J* = 8.4 Hz, 1H), 7.32 (t, *J* = 8.4 Hz, 1H), 7.25 (d, *J* = 16.3 Hz, 1H), 7.20–7.12 (m, 2H), 6.51 (s, 1H), 3.48–3. 41(m, 2H), 2.57 (t, *J* = 4.4 Hz, 2H), 2.37 (s, 6H), 1.97–1.90 (m, 2H). ^13^C-NMR (101 MHz, CDCl_3_): δ 157.13, 151.21, 148.19, 137.22, 129.39, 128.06, 127.69, 126.97, 125.85, 124.98, 123.45, 122.62, 121.09, 120.94, 120.68, 119.76, 115.44, 112.10, 95.36, 54.70, 45.72, 44.15, 25.45. Purity: 98.5% by HPLC. HRMS (ESI): Cacld for [M + H]^+^ (C_24_H_27_N_4_) requires *m/z* 371.2230, found 371.2245.

*(E)-N^1^-(2-(4-(Diethylamino)styryl)quinolin-4-yl)-N^2^,N^2^-dimethylethane-1,2-diamine* (**6c_1_**)

Compound **5c** was reacted with 4-diethylaminobenzaldehyde following the general procedure A to afford product **6c_1_** as an orange solid in 77% yield. mp. 145.2–147.1 °C. ^1^H-NMR (400 MHz, CDCl_3_): δ 7.98 (d, *J* = 8.4 Hz, 1H), 7.75 (d, *J* = 8.1 Hz, 1H), 7.60–7.48 (m, 4H), 7.36 (t, *J* = 4.0 Hz, 1H), 7.10 (d, *J* = 16.1 Hz, 1H), 6.75–6.59 (m, 3H), 3.40 (dd, *J* = 14.0, 7.1 Hz, 6H), 2.73 (t, *J* = 5.8 Hz, 2H), 2.33 (s, 6H), 1.20 (t, *J* = 7.0 Hz, 6H). ^13^C-NMR (101 MHz, CDCl_3_): δ 157.50, 149.85, 148.63, 147.95, 133.62, 129.29, 129.13, 128.72, 124.90, 124.07, 123.75, 119.66, 118.36, 111.52, 96.30, 57.24, 45.13, 44.40, 40.08, 12.70. Purity: 99.4% by HPLC. HRMS (ESI): Cacld for [M + H]^+^ (C_25_H_33_N_4_) requires *m/z* 389.2700, found 389.2712.

*(E)-N^1^-(2-(4-(Dimethylamino)styryl)quinolin-4-yl)-N^2^,N^2^-dimethylethane-1,2-diamine* (**6c_2_**)

Compound **5c** was reacted with 4-dimethylaminobenzaldehyde following the general procedure A to afford product **6c_2_** as an orange-red solid in 79% yield. mp. 148.9–149.6 °C. ^1^H-NMR (400 MHz, CDCl_3_): δ 8.02 (d, *J* = 8.5 Hz, 1H), 7.76 (t, *J* = 8.1 Hz, 1H), 7.60–7.53 (m, 2H), 7.51 (d, *J* = 8.7 Hz, 2H), 7.34 (t, *J* = 7.6 Hz, 1H), 7.26 (t, *J* = 4.3 Hz, 1H), 7.12 (d, *J* = 16.4Hz, 1H), 6.70 (d, *J* = 8.2Hz, 2H), 6.59 (s, 1H), 3.39 (s, 2H), 2.99 (s, 6H), 2.71 (t, *J* = 5.9 Hz, 2H), 2.32 (s, 6H). ^13^C-NMR (101 MHz, CDCl_3_): δ 157.27, 149.60, 148.04, 147.43, 133.79, 129.68, 129.18, 128.56, 124.73, 124.48, 123.42, 119.27, 118.29, 113.76, 98.04, 56.25, 45.31, 43.21, 40.29. Purity: 96.6% by HPLC. HRMS (ESI): Cacld for [M + H]^+^ (C_23_H_29_N_4_) requires *m/z* 361.2387, found 361.2399.

*(E)-N-Butyl-2-(4-(diethylamino)styryl)quinolin-4-amine* (**6d_1_**)

Compound **5d** was reacted with 4-diethylaminobenzaldehyde following the general procedure A to afford product **6d_1_** as a yellow solid in 80% yield. mp. 172.5–173.8 °C. ^1^H-NMR (400 MHz, CDCl_3_): δ 8.13 (s, 1H), 7.91 (d, *J* = 8.2 Hz, 1H), 7.75 (d, *J* = 8.0 Hz, 1H), 7.51 (m, 3H), 7.29 (t, *J* = 8.2 Hz, 1H), 7.07 (d, *J* = 16.2 Hz, 1H), 6.64 (d, *J* = 8.6 Hz, 2H), 6.53 (s, 1H), 3.40–3.34 (m, 4H), 3.27 (q, *J* = 6.7 Hz, 2H), 1.54–1.43 (m, 2H), 1.37–1.29 (m, 2H), 1.17 (t, *J* = 7.0 Hz, 6H), 0.89 (t, *J* = 7.3 Hz, 3H). ^13^C-NMR (101 MHz, CDCl_3_): δ 157.17, 150.60, 149.79, 146.44, 135.33, 133.74, 129.33, 129.20, 128.49, 125.03, 123.88, 119.06, 118.04, 112.25, 96.30, 43.03, 40.37, 31.15, 20.40, 14.19, 13.92. Purity: 98.8% by HPLC. HRMS (ESI): Cacld for [M + H]^+^ (C_25_H_32_N_3_) requires *m*/*z* 374.2591, found 374.2606.

*(E)-N-Butyl-2-(4-(dimethylamino)styryl)quinolin-4-amine* (**6d_2_**)

Compound **5d** was reacted with 4-dimethylaminobenzaldehyde following the general procedure A to afford product **6d_2_** as an orange solid in 82% yield. mp. 165.3–167.1 °C. ^1^H-NMR (400 MHz, CDCl_3_): δ 7.96 (d, *J* = 2.3 Hz, 1H), 7.78–7.68 (m, 1H), 7.61–7.50 (m, 3H), 7.37–7.28 (m, 1H), 7.24 (d, *J* = 8.0 Hz, 1H), 7.10 (d, *J* = 16.2 Hz, 1H), 6.70 (d, *J* = 8.0 Hz, 2H), 6.55 (s, 1H), 3.39 (dd, *J* = 12.3, 6.9 Hz, 2H), 3.02 (s, 6H), 1.53–1.48 (m, 2H), 1.35–1.28 (m, 2H), 0.87 (t, *J* = 7.3 Hz, 3H). ^13^C-NMR (101 MHz, CDCl_3_): δ 158.12, 155.18, 147.76, 146.34, 136.29, 130.93, 130.50, 129.23, 128.78, 124.62, 124.18, 123.69, 119.44, 118.83, 112.19, 111.74, 103.93, 51.96, 40.31, 31.25, 20.38, 13.88. Purity: 98.3% by HPLC. HRMS (ESI): Cacld for [M + H]^+^ (C_23_H_28_N_3_) requires *m/z* 346.2278, found 346.2296.

*(E)-2-(4-(Diethylamino)styryl)-N-isobutylquinolin-4-amine* (**6e_1_**)

Compound **5e** was reacted with 4-diethylaminobenzaldehyde following the general procedure A to afford product **6e_1_** as a pale yellow solid in 85% yield. mp. 170.2–171.7 °C. ^1^H-NMR (400 MHz, CDCl_3_): δ 7.96 (d, *J* = 8.3 Hz, 1H), 7.66 (t, *J* = 8.2 Hz, 1H), 7.61–7.55 (m, 2H), 7.53–7.48 (m, 1H), 7.35 (t, *J* = 7.9 Hz, 1H), 7.22 (d, *J* = 8.7 Hz, 2H), 7.08 (d, *J* = 16.2 Hz, 1H), 6.79 (d, *J* = 12.3 Hz, 1H), 6.67 (d, *J* = 8.8 Hz, 1H), 5.00 (s, 1H), 3.32 (q, *J* = 7.0 Hz, 4H), 2.83 (t, *J* = 6.2 Hz, 2H), 1.78–1.70 (m, 1H), 1.13 (t, *J* = 7.1 Hz, 6H), 0.86 (d, *J* = 6.4 Hz, 6H). ^13^C-NMR (101 MHz, CDCl_3_): δ 158.18, 149.89, 148.93, 147.28, 134.17, 130.73, 129.49, 129.20, 128.79, 128.03, 124.19, 119.06, 117.94, 110.97, 100.09, 50.77, 44.28, 27.48, 20.30, 12.63 ppm. Purity: 97.7% by HPLC. HRMS (ESI): Cacld for [M + H]^+^ (C_25_H_32_N_3_) requires *m/z* 374.2591, found 374.2606.

*(E)-2-(4-(Dimethylamino)styryl)-N-isobutylquinolin-4-amine* (**6e_2_**)

Compound **5e** was reacted with 4-dimethylaminobenzaldehyde following the general procedure A to afford product **6e_2_** as an orange solid in 83% yield. mp. 171.8–173.2 °C. ^1^H-NMR (400 MHz, CDCl_3_): δ 7.97 (d, *J* = 8.3 Hz, 1H), 7.77 (d, *J* = 7.7 Hz, 1H), 7.58–7.49 (m, 4H), 7.32 (t, *J* = 7.6 Hz, 1H), 7.14 (d, *J* = 16.2 Hz, 1H), 6.68 (d, *J* = 8.4 Hz, 2H), 6.55 (d, *J* = 8.6 Hz, 1H), 3.21 (t, *J* = 6.0 Hz, 2H), 2.98 (s, 6H), 2.14–2.03 (m, 1H), 1.08 (d, *J* = 6.6 Hz, 6H). ^13^C-NMR (101 MHz, CDCl_3_): δ 156.91, 150.22, 148.79, 147.57, 134.94, 129.57, 129.17, 129.01, 128.62, 127.40, 124.33, 118.98, 117.64, 112.22, 98.27, 51.21, 43.98, 28.19, 19.95. Purity: 97.9% by HPLC. HRMS (ESI): Cacld for [M + H]^+^ (C_23_H_28_N_3_) requires *m/z* 346.2278, found 346.2292.

### 3.3. Pharmacological Assay

#### 3.3.1. ThT Assay 

All ThT experiments were performed according to our previously published methods [40]. Aβ_1–42_ (Sigma, St. Louis, MO, USA; counterion, NaOH) stock solution was prepared by dissolving Aβ_1–42_ peptide in ammonium hydroxide (1% *v/v*), and diluting with 20 mM phosphate buffer (pH 7.4) to 100 μM. The tested compounds were firstly dissolved in DMSO at a concentration of 10 mM, and then diluted with 20 mM phosphate buffer (pH 7.4) to 40 μM.

For the inhibition of self-induced Aβ_1–42_ aggregation [40]. Aβ_1–42_ peptide (2 μL, 50 μM, final concentration) with the tested compound (2 μL, 20 μM, final concentration) or 20 mM phosphate buffer (2 μL) was incubated in 20 mM phosphate buffer (pH 7.4) at 37 °C for 72 h. After incubation, the samples were diluted to a final volume of 36 μL with 50 mM glycine-NaOH buffer (pH 8.5) containing 5 μM Thioflavin T. Fluorescence signal was measured (excitation wavelength 450 nm, emission wavelength 485 nm, and slit widths set to 5 nm) on a monochromator-based multimode microplate reader (INFINITE M1000, TECAN, Hombrechtikon, Switzerland), adapted for 384-well microtiter plates. The fluorescence intensities were recorded, and the percent inhibition of Aβ_1–42_ aggregation was calculated with the following equation: (1 − *I*_Fi_/*I*_Fc_) × 100%, in which *I*_Fi_ and *I*_Fc_ are the fluorescence intensities obtained for absorbance in the presence and absence of the compounds after subtracting the background, respectively. Each compound was examined in triplicate. 

For the disaggregation of self-induced Aβ_1–42_ fibrils [52]. the Aβ_1–42_ stock solution (2 μL, 50 μM, final concentration) was incubated at 37 °C for 24 h. Then, the 40 μM tested compound (2 μL) or 20 mM phosphate buffer (2 μL) was added and incubated at 37 °C for another 24 h. After incubation, the sample was diluted to a final volume of 40 μL with 50 mM glycine-NaOH buffer (pH 8.0) containing thioflavin T (5 μM). The detection method was the same as above.

#### 3.3.2. Antioxidant Activity Assay In Vitro

The antioxidant activities of the compounds in vitro were determined by the oxygen radical absorbance capacity fluorescein (ORAC-FL) assay [44,45]. The tested compounds and (±)-6-hydroxy-2,5,7,8-tetramethylchroman-2-carboxylic acid (Trolox) were dissolved in DMSO at a concentration of 10 mM, and then diluted with 75 mM potassium phosphate buffer (pH 7.4) to 50 μM and 10 μM for use. The fluorescein (FL) stock solution (3.4 mM) was diluted with the same buffer to 136 nM. All the assays were conducted with 75 mM potassium phosphate bufffer (pH 7.4), and the final reaction mixture was 200 μL. 

The mixture of compounds (or buffer) (20 μL) and fluorescein (FL, 120 μL) were incubated for 15 min at 37 °C in a black 96-well plate. Then 2,2′-azobis-(amidino-propane)-dihydrochloride (AAPH) solution (60 μL, 40 mM) was rapidly added to the reaction mixture. The fluorescence was recorded every 5 min for 240 min at 485 nm (excitation wavelength) and 535 nm (emission wavelength) (Varioskan Flash Multimode Reader, Thermo Scientific, Waltham, MA, USA), and each sample was prepared and measured for three times independently. Antioxidant curves (fluorescence versus time) were normalized to the curve of the blank (without antioxidant), and the area under the fluorescence decay curve (AUC) was calculated by the following equation:

(1) $$\mathrm{AUC}\;=\;1\;+\;\sum_{i=1}^{i=120}\left(f_{i}/f_{0}\right)$$

where *f*_0_ is the initial fluorescence at 0 min and *f_i_* is the fluorescence at time *i*. The net AUC for a sample was calculated using the expression net AUC = AUC_sample_ − AUC_blank_, The ORAC-FL value of each sample was calculated using the equation of net AUC_sample_/(AUC_Trolox_ – AUC_blank_), which was expressed as Trolox equivalents.

#### 3.3.3. Antioxidant Activity in SH-SY5Y Cells

SH-SY5Y cells were maintained in Dulbecco’s modified Eagle’s medium (DEME) and supplemented with 1 mM glutamine, 10% fetal calf serum, 100 U/mL penicillin, and 100 μg/mL streptomycin. The cultures were maintained at 37 °C in a humidified incubator containing 5% CO_2_ and were passaged twice weekly. 

The antioxidant activity of the compounds in SH-SY5Y cells were tested with the fluorescent probe (2′,7′-dichlorofluorescein diacetate, DCFH-DA) as reported with some variation [46]. SH-SY5Y cells were sub-cultured in 96-well plates at a seeding density of 1 × 10^4^ cells per well for 24 h, then the medium was removed, the tested compounds (2.5 μM) was added and incubated for another 24 h at 37 °C. After that, the cells were washed with PBS and incubated with 5 μM DCFH-DA in PBS at 37 °C in 5% CO_2_ for 30 min. Then DCFH-DA was removed, the cells were washed three times and incubated with 0.1 mM *t*-BuOOH in PBS for 30 min. After the incubation, the cell fluorescence from each well was measured at 485 nm excitation and 535 nm emission with a monochromator based multimode microplate reader (INFINITE M1000). 

#### 3.3.4. Metal Chelation 

The metal chelation of compound **6b_1_** was tested in ethanol using a UV-vis spectrophotometer (Shimadzu UV-2450PC) with wavelength ranging from 200 to 700 nm [47,48]. The absorption spectra of compound **6b_1_** (10 μM, final concentration) alone or in the presence of metal ions (20 μM, final concentration) after incubation for 30 min at room temperature, was recorded at room temperature in a 1 cm quartz cell. Each sample was repeated for at least three times.

Experiments for the determination of metal ion binding selectivity were carried as follows [48]. 20.0 mM stock solutions of CuSO_4_, ZnSO_4_, FeSO_4_, CaCl_2_, MgCl_2_, NaCl and KCl in MQ water were prepared and then diluted to 1.0 mM using ethanol. 2.0 mM solution of compound **6b_1_**, CQ and resveratrol in ethanol were freshly prepared prior to use. 10 μL solutions of the compounds treated with 10 μL of ZnSO_4_, FeSO_4_, CaCl_2_, MgCl_2_, NaCl and KCl. Spectra were recorded after 10 min incubation at 25 °C. The metal binding selectivity was assessed by then adding 10 μL of CuSO_4_ solution and incubating for 10 additional min at 25 °C. Selectivity was quantified by comparing and normalizing the absorbance values of the maximum in each case with the absorbance of the solution at the same wavelength after addition of CuSO_4_.

For binding stoichiometry assay, A fixed amount of compound **6b_1_** (20 μM) was mixed with growing amounts of copper ion (0–20 μM) and tested the difference UV-vis spectra to investigate the ratio of ligand/metal in the complex.

#### 3.3.5. Inhibition of Cu^2+^-Induced Aβ_1–42_ Aggregation

Aβ was diluted in 20 μM HEPES (pH 6.6) with 150 μM NaCl. The mixture of the peptide (10 μL, 20 μM, final concentration) with or without copper (10 μL, 20 μM, final concentration) and the test compound (10 μL, 40 μM, final concentration) was incubated at 37 °C for 24 h. The 30 μL of the sample was diluted to a final volume of 200 μL with 50 mM glycine-NaOH buffer (pH 8.0) containing thioflavin T (5 μM) [49]. The detection method was the same as that of self-induced Aβ aggregation experiment.

#### 3.3.6. Cytotoxicities of the Compounds on SH-SY5Y Cells

The cytotoxicities of the compounds were evaluated using the MTT assay [50]. SH-SY5Y cells were subcultured in 96-well plates at a seeding density of 1 × 10^4^ cells per well. After 24 h, the medium was removed and treated with different concentrations of tested compounds for 24 h at 37 °C. The survival of cells was determined with MTT assay. Then 80 μL of medium and 20 μL of MTT (0.5 mg/mL, final concentration) were added to each well and incubated for another 4 h. After the removal of MTT, the formazan crystals were dissolved in DMSO. The amount of formazan was measured using a microculture plate reader at the wavelength of 570 nm. Each concentration was performed in triplicate.

#### 3.3.7. TEM Assay 

Aβ_1–42_ peptide (Sigma) was dissolved in 20 mM phosphate buffer (pH 7.4) at 4 °C to 100 μM before use. 

For the inhibition of self-induced Aβ_1–42_ aggregation experiment [51,52]. it was incubated in the presence and absence of compound **6b_1_** and resveratrol at 37 °C for 24 h; For the disaggregation of self-induced Aβ_1–42_ fibril experiment [52]. Aβ_1–42_ was incubated at 37 °C for 24 h. Then, 40 μM tested compound was added and incubated at 37 °C for another 24 h. The final concentrations of Aβ_1–42_ and the compounds were 50 μM and 20 μM, respectively. After incubation, aliquots (5 μL) of the samples were placed on a carbon-coated copper/rhodium grid for 2 min at room temperature. Each grid was stained with uranyl acetate (1%, 5 μL) for 2 min. Excess staining solution was removed and the specimen was transferred for imaging with transmission electron microscopy (JEOL JEM-1400, Tokyo, Japan). 

#### 3.3.8. Aβ_1–42_-Induced Neurotoxicity

SH-SY5Y cells were seeded in 96-well plates (1 × 10^4^ cells per well) and exposed to Aβ_1–42_ (20 μM) in the presence of compound **6b_1_** at the increasing doses (0, 5, 10 and 20 μM). After 24 h and 48 h, cell viability was measured by the MTT assay [53,54]. 

#### 3.3.9. Step-Down Type Passive Avoidance Test

A step-down passive avoidance test was used to assess learning and memory in mice [55,56]. The apparatus (Shanghai XinRuan, Shanghai, China) consisted of two identically sized compartments, with a guillotine door to separate light and dark. Illumination was available in the light box through LED lights at 250 lux. The mice underwent two separate trials: a training trial and a test trial 24 h later. For training trial, mice were initially placed in the light compartment and were allowed to explore the environment freely for 5 min so that they can be familiar with the environment. Then the door was opened, the mice tried to enter the dark compartment since they preferred to stay in dark place. As soon as the mice came into the dark compartment, an electrical shock was delivered through the steel rods. The training lasted for 5 min. Mice which did not enter the dark compartment within 180 s were excluded from the test. The test trial was preformed after 24 h. The mice were placed into the light compartment and the door was opened. The time that the mouse spent to enter the dark compartment was recorded as the latency. The numbers that the mouse entered the dark compartment during 5 min were measured as error numbers. If the mouse did not cross the door, the latency was identified as 300 s. 

## 4. Conclusions

In summary, a series of 4-flexible amino-2-arylethenylquinoline derivatives was synthesized and characterized as multi-target anti-Alzheimer agents based on our previous study. Most compounds displayed high effective inhibitory potencies against Aβ_1–42_ aggregation and antioxidant activity. The structure-activity relationship was summarized, which confirmed the importance of diamino substitution group at the 4-postion of the quinoline ring for Aβ_1–42_ aggregation inhibition. In addition, the substituent group featuring a *N*,*N*-dimethylaminoalkylamino moiety at the 4-postion of the quinoline scaffold displayed significantly increased activity. The optimal candidate compound **6b_1_** also displayed metal-chelating ability and 85.8% inhibition of Cu^2+^-induced Aβ aggregation, good disaggregation of Aβ_1–42_ fibrils generated by self-induced Aβ_1–42_ aggregation. Moreover, it exhibited low toxicity to SH-SY5Y cells and a significant effect on the protection of neuronal cells against Aβ_1–42_-induced cytotoxicity in SH-SY5Y cells. Most importantly, compound **6b_1_** could significantly prolong the latency and reduce number of errors in the step-down passive avoidance test. Unfortunately, its inhibitory activity toward AChE and BuChE was weak (data not shown). Such excellent properties highlight compound **6b_1_** as a potential lead compound for new multitarget drug development in the treatment of AD. 

## Figures and Tables

**Table 1:**
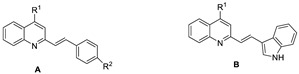
Inhibition of self-induced Aβ_1–42_ aggregation, ORAC values of the target compounds.

Compound	General Structure	R^1^	R^2^	Aβ_1–42_ Aggregation Inhibition ^a^ (%)	ORAC ^b^	
					5 μM	1 μM
**6a_1_**	A	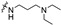	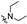	90.2 ± 1.3	6.85 ± 0.05	5.92 ± 0.03
**6a_2_**	A	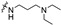	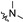	88.5 ± 1.5	6.28 ± 0.08	5.74 ± 0.09
**6a_3_**	B	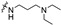	_	84.3 ± 2.1	1.73 ±0.07	1.26 ± 0.06
**6a_4_**	A	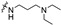	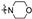	87.9 ± 1.1	4.52 ± 0.03	4.16 ± 0.04
**6b_1_**	A	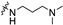	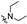	95.3 ± 1.2	6.54 ± 0.07	6.23 ± 0.05
**6b_2_**	A	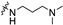	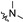	92.1 ± 1.3	5.88 ± 0.01	5.61 ± 0.09
**6b_3_**	B	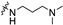	_	87.2 ± 2.1	4.21 ± 0.08	3.96 ± 0.08
**6c_1_**	A	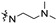	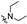	85.1 ± 1.4	3.24 ± 0.10	2.79 ± 0.04
**6c_2_**	A	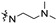	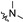	83.9 ± 2.4	3.53 ± 0.07	3.28 ± 0.09
**6d_1_**	A	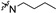	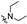	80.5 ± 1.4	1.56 ± 0.05	1.38 ± 0.02
**6d_2_**	A	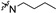	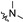	79.2 ± 2.3	0.95 ± 0.09	0.75 ± 0.06
**6e_1_**	A	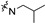	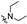	77.9 ± 1.3	1.92 ± 0.11	1.71 ± 0.05
**6e_2_**	A	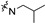	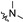	77.5 ± 1.5	1.78 ± 0.05	1.68 ± 0.06
**4b_1_**	A	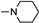	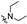	83.5 ± 1.4	3.91 ± 0.11	3.70 ± 0.11
**4b_2_**	A	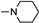	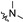	81.2 ± 0.8	3.12 ± 0.10	2.91 ± 0.11
**Res**	_	_	_	77.2 ± 1.1	5.21 ± 0.50	5.10 ± 0.41
**Cur**	_	_	_	49.3 ± 1.2	2.61 ± 0.12	2.11 ± 0.61

^a^ The Thioflavin T fluorescence method was used. The maximum percentage inhibitions of aggregation (means ± SD for these experiments) were found at the inhibitors’ concentration of 20 μM. ^b^ Data were expressed as μmol of Trolox equivalents/μmol of tested compounds. The concentration of the tested compounds was 5 μM and 1 μM.

**Table 2:** The IC_50_ values of compound **6a_1_, 6b_1_, 6b_2_,** and resveratrol against self-induced Aβ_1–42_ aggregation.

Compound	IC_50_ (μM) ^a^
**6a_1_**	7.8 ± 0.4
**6b_1_**	4.5 ± 0.7
**6b_2_**	6.1 ± 0.8
**Res**	11.8 ± 0.2

^a^ The Thioflavin T fluorescence method was used, the IC_50_ (μM) values shown are the mean ± SD of three experiments.

## Supplementary Materials

Supplementary data (^1^H-NMR, ^13^C-NMR, HRMS and HPLC spectra) associated with this article are available online.
