# Supplementary material for: Preparation of 4-Flexible Amino-2-Arylethenyl-Quinoline Derivatives as Multi-Target Agents for the Treatment of Alzheimer’s Disease
Source: Molecules. 2018 Nov 27;23(12):3100. doi: 10.3390/molecules23123100 (PMC6321145; doi:10.3390/molecules23123100)
Supplement: Supplementary file 1 [file molecules-23-03100-s001.pdf]

Supporting Information

Preparation of Novel 4-Flexible amino-2-arylethenylquinoline Derivatives as Multitarget Agents for the Treatment of Alzheimer's Disease

Xiao-Qin Wang*, Chu-Ping Zhao, Long-Cheng Zhong, De-Ling Zhu, De-Hao Mai, Mei-Gui Liang, Ming-Hua He*

School of Pharmacy, Guangdong Medical University (GDMU), No.1 Xincheng road, Songshan Lake, Dongguan, Guangdong Province (P.R. China)

E-mail: wangxqgdmu@hotmail.com; cpuhmh@163.com

The representative ^{1}H NMR, ^{13}C NMR spectra, HPLC and HRMS spectra of the target compounds.

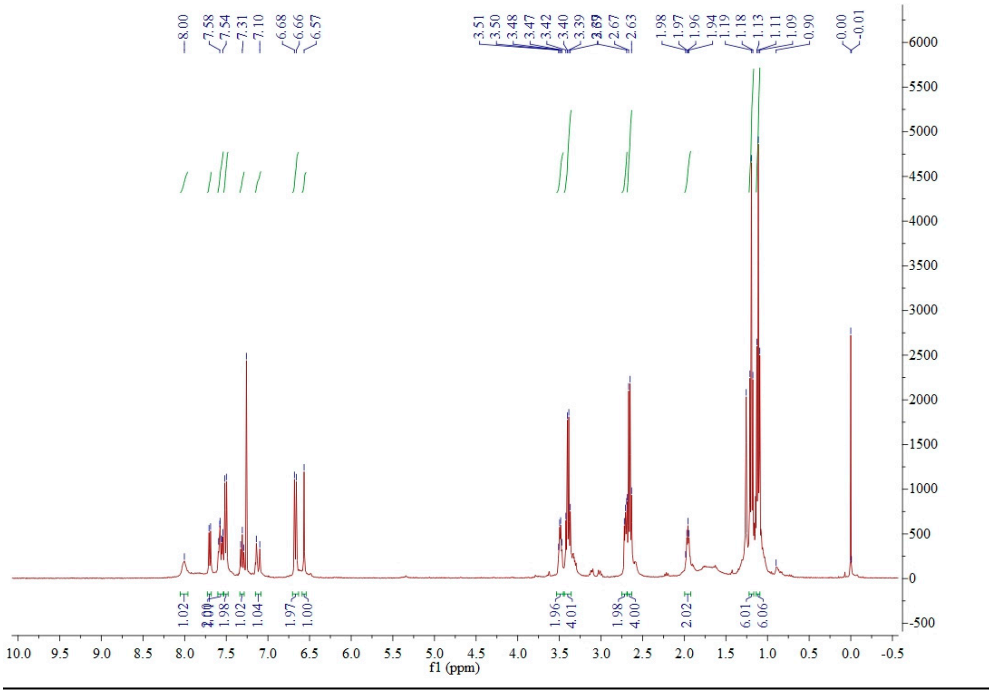

^{1}H NMR spectrum of **6a1**

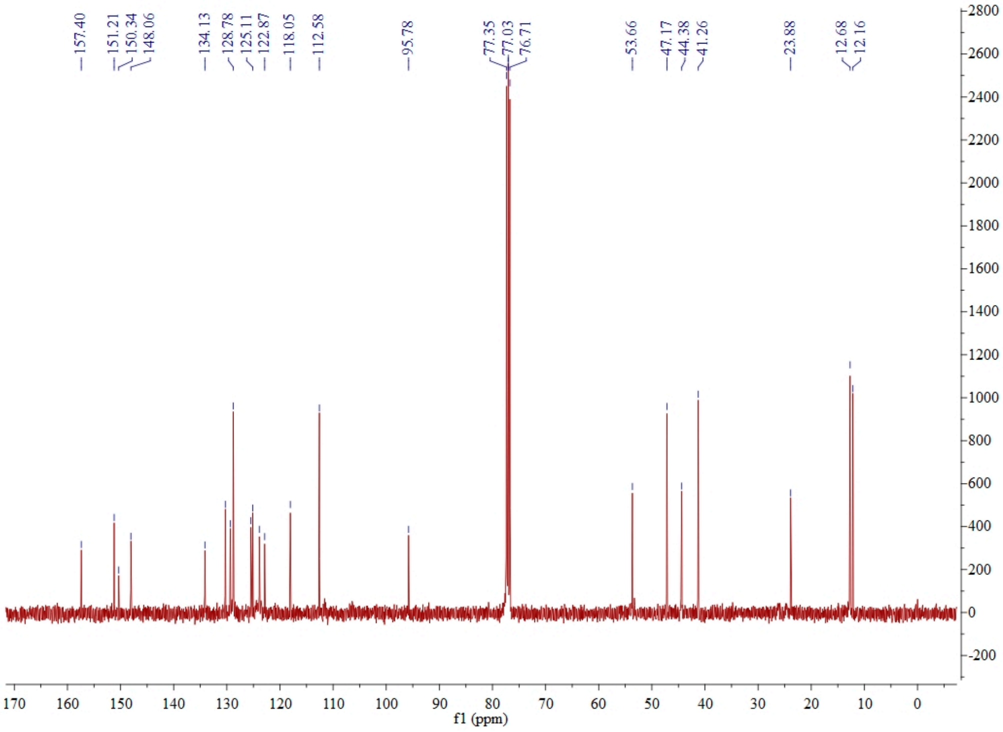

^{13}C NMR spectrum of **6a1**

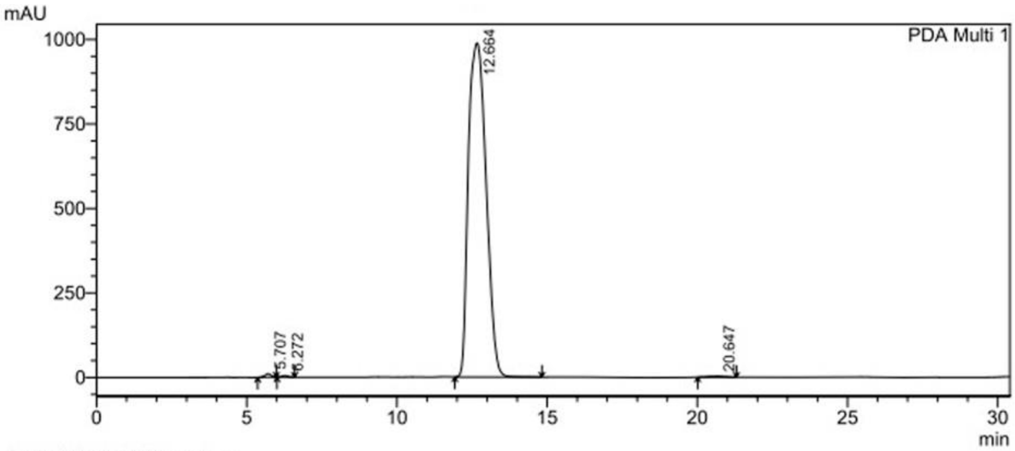

1 PDA Multi 1/254nm 4nm

PeakTable

PDA Ch1 254nm 4nm

Peak#	Ret. Time	Area	Height	Area %	Height %
1	5.707	135319	9934	0.331	0.988
2	6.272	63128	4598	0.154	0.457
3	12.664	40618406	988255	99.245	98.282
4	20.647	110647	2747	0.270	0.273
Total		40927500	1005533	100.000	100.000

HPLC spectrum of **6a**1

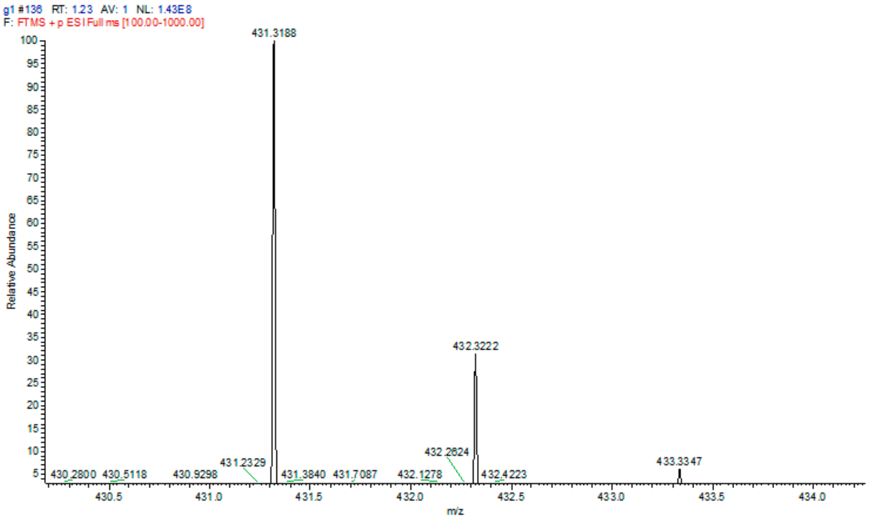

Idx	Formula	RDB	Delta mmu
1	$C_{28}H_{39}N_4$	11.5	1.896

Caculated 431.3169 Found 431.3188

HRMS spectra of **6a**1

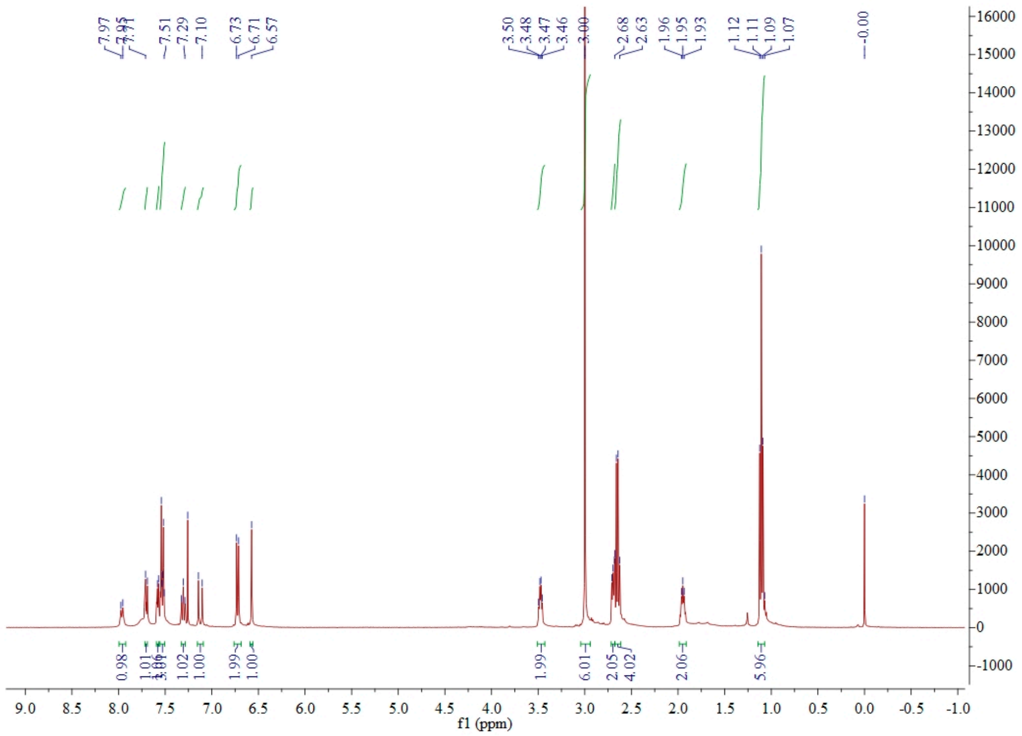

^{1}H NMR spectrum of **6a2**

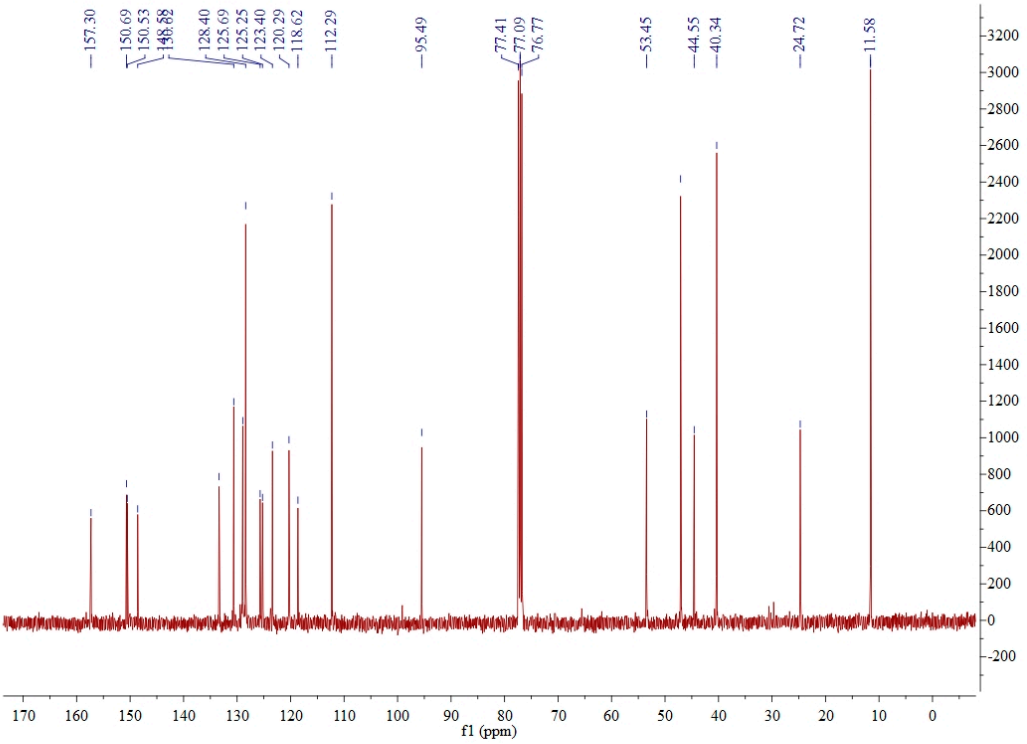

^{13}C NMR spectrum of **6a2**

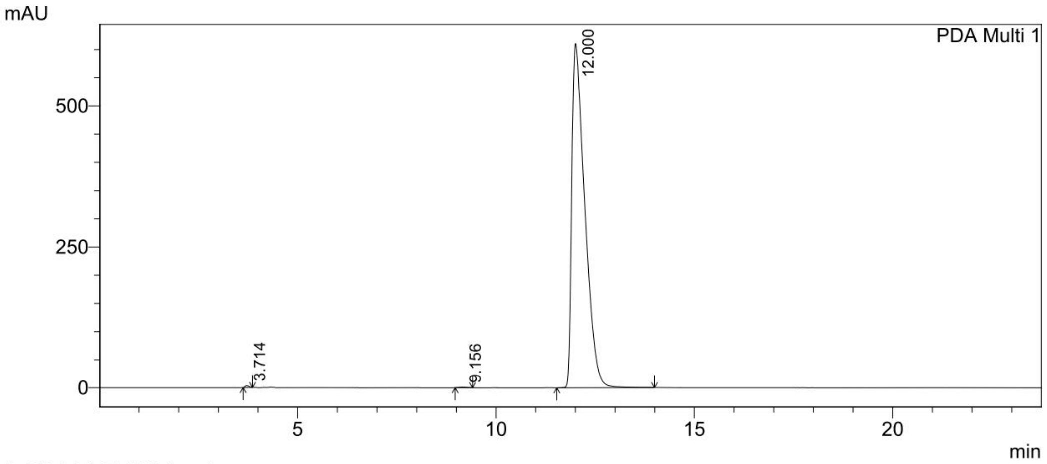

1 PDA Multi 1/254nm 4nm

PeakTable

PDA Ch1 254nm 4nm

Peak#	Ret. Time	Area	Height	Area %	Height %
1	3.714	23334	3522	0.167	0.573
2	9.156	13924	1050	0.099	0.171
3	12.000	13969692	610445	99.734	99.257
Total		14006950	615017	100.000	100.000

HPLC spectrum of **6a**$_2$

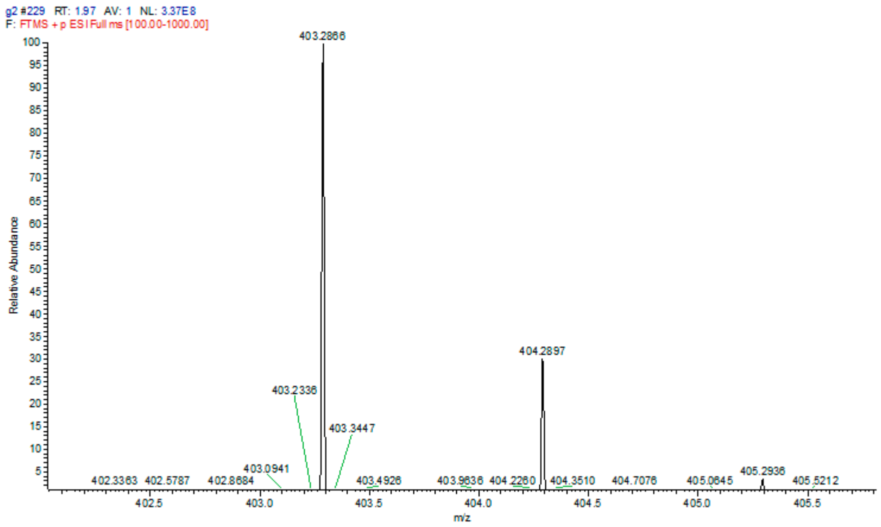

Idx	Formula	RDB	Delta mmu
1	$C_{26}H_{35}N_4$	11.5	0.996

Caculated 403.2856 Found 403.2866

HRMS spectra of **6a**$_2$

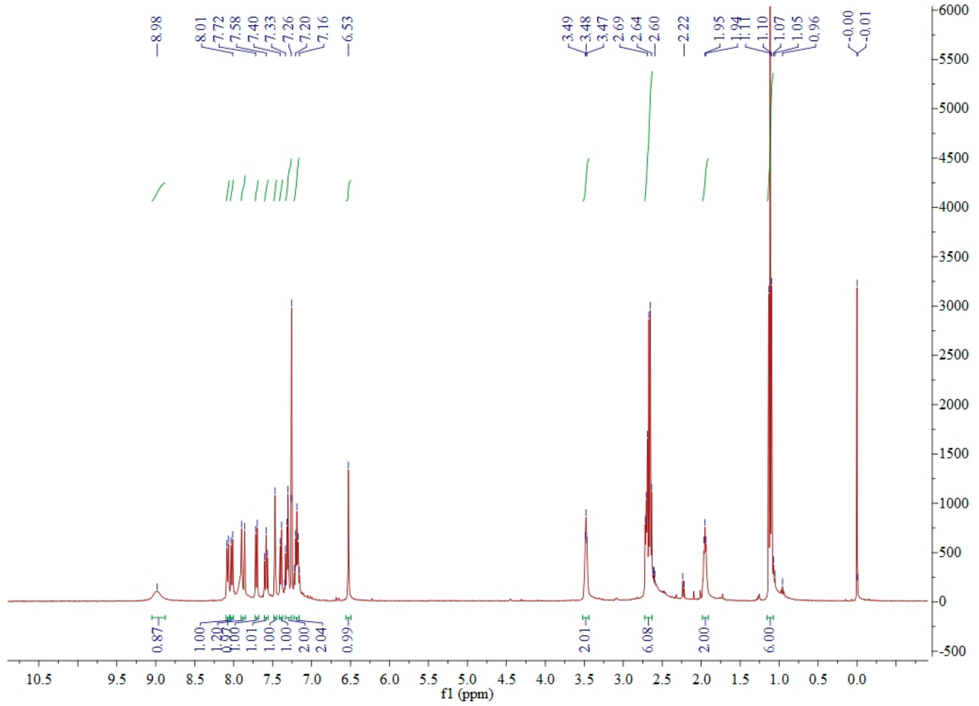

^{1}H NMR spectrum of **6a**3

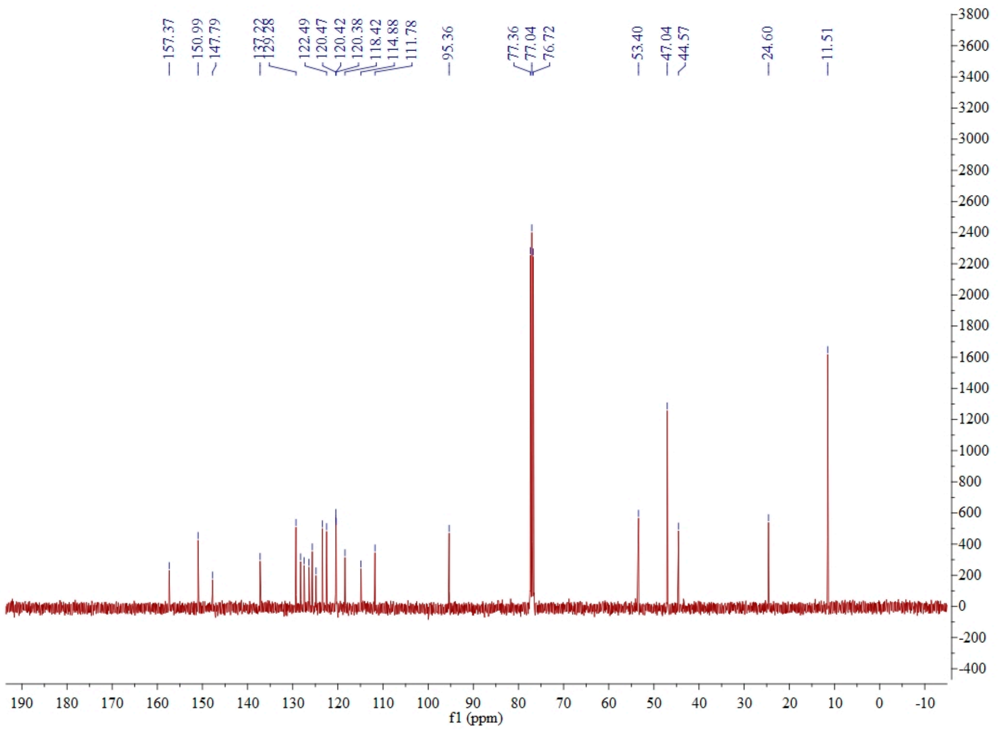

^{13}C NMR spectrum of **6a**3

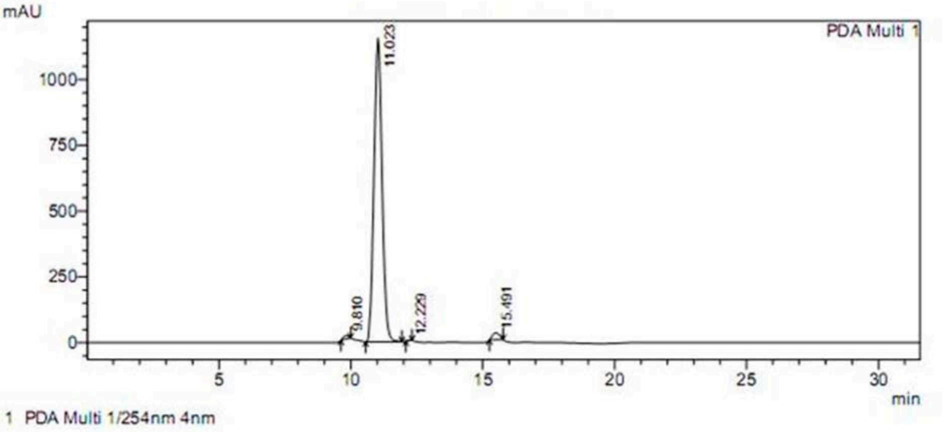

PeakTable

PDA Ch1 254nm 4nm

Peak#	Ret. Time	Area	Height	Area %	Height %
1	9.810	191832	13552	0.765	1.132
2	11.023	24373177	1154709	97.198	96.473
3	12.229	22654	2414	0.090	0.202
4	15.491	488049	26246	1.946	2.193
Total		25075711	1196920	100.000	100.000

HPLC spectrum of **6a**3

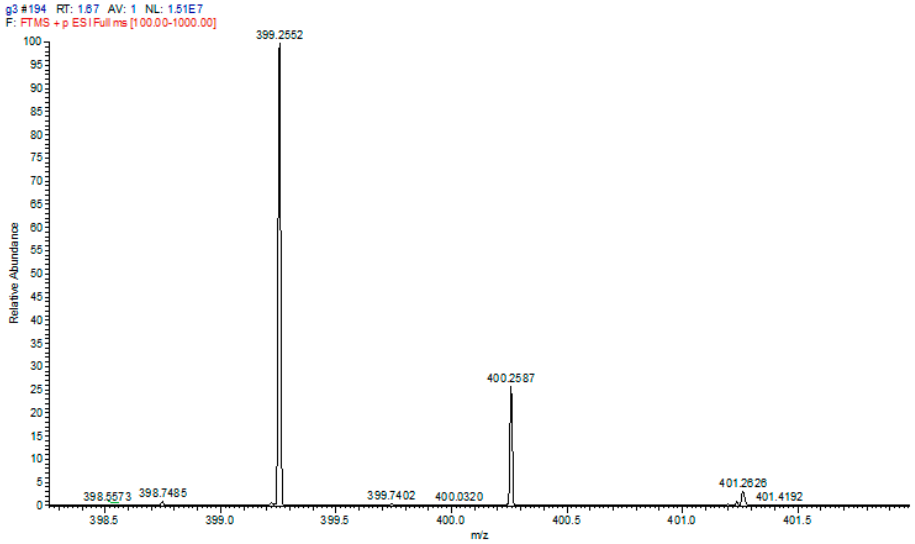

Idx	Formula	RDB	Delta mmu
1	$C_{26}H_{31}N_4$	13.5	0.926

Caculated 399.2543 Found 399.2552

HRMS spectra of **6a**3

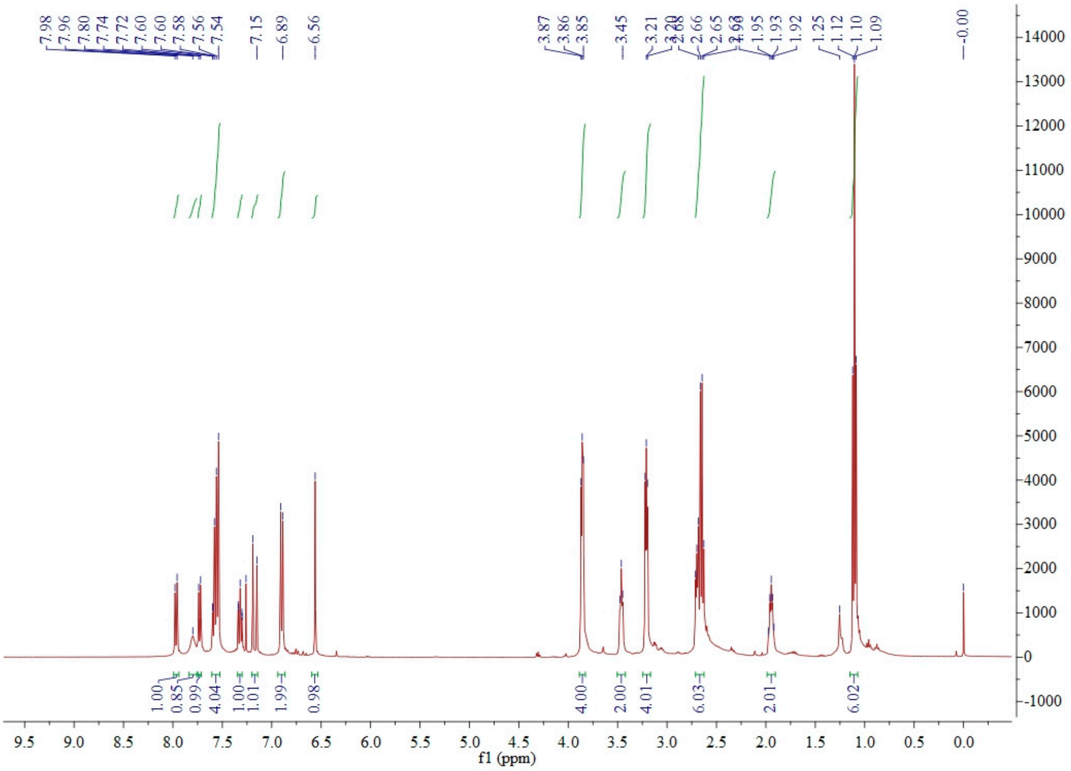

^{1}H NMR spectrum of **6a**$_4$

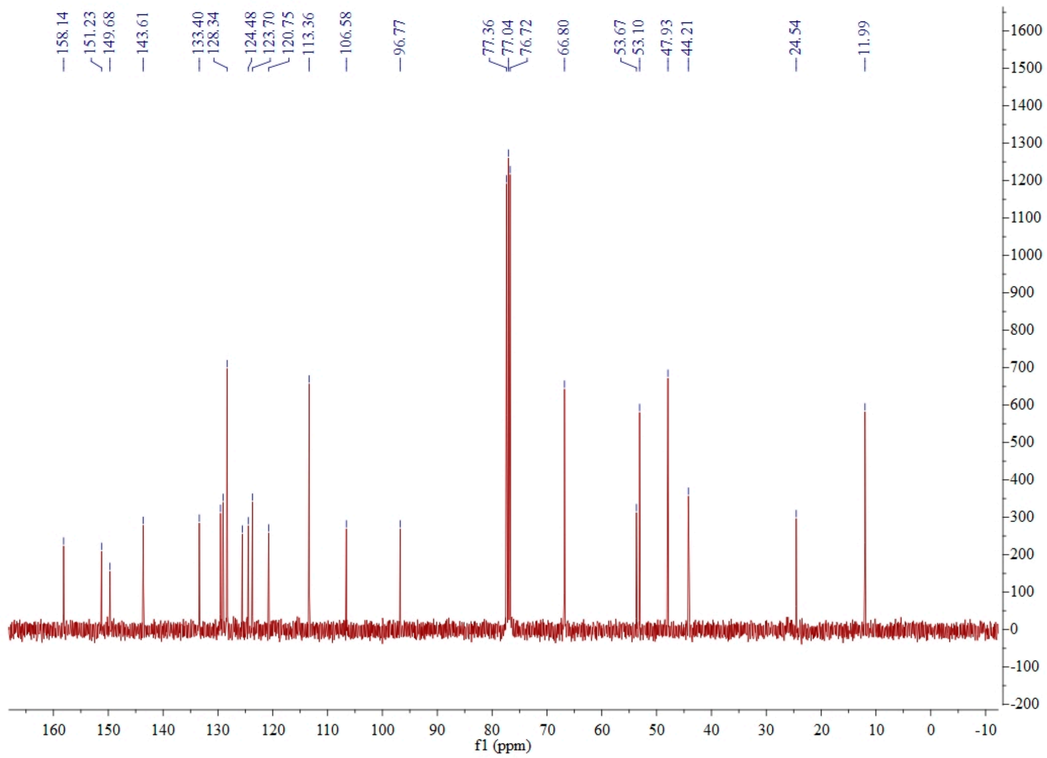

^{13}C NMR spectrum of **6a**$_4$

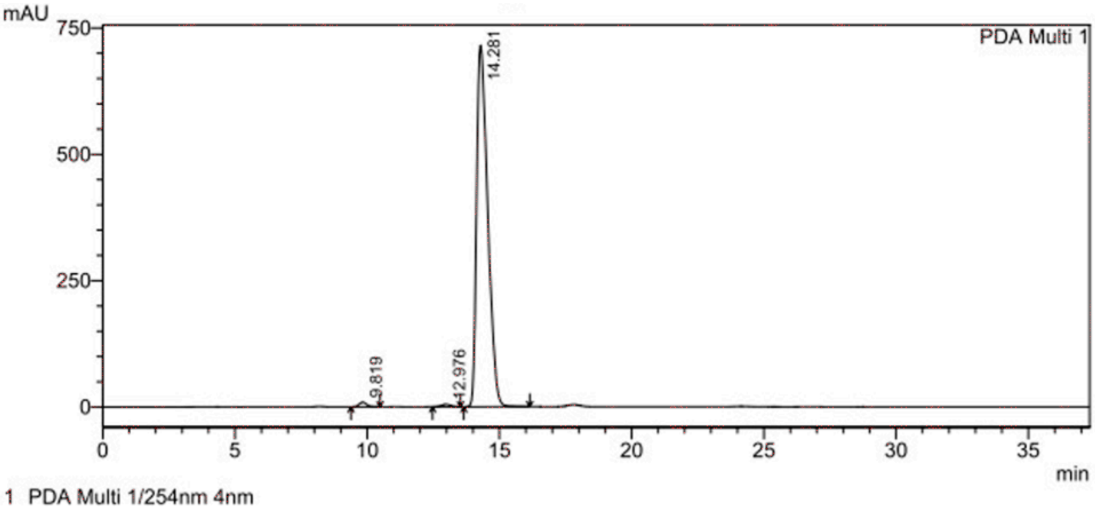

PeakTable

PDA Ch1 254nm 4nm

Peak#	Ret. Time	Area	Height	Area %	Height %
1	9.819	177853	9009	0.843	1.235
2	12.976	111004	4849	0.526	0.665
3	14.281	20806314	715474	98.631	98.100
Total		21095171	729332	100.000	100.000

HPLC spectrum of **6a4**

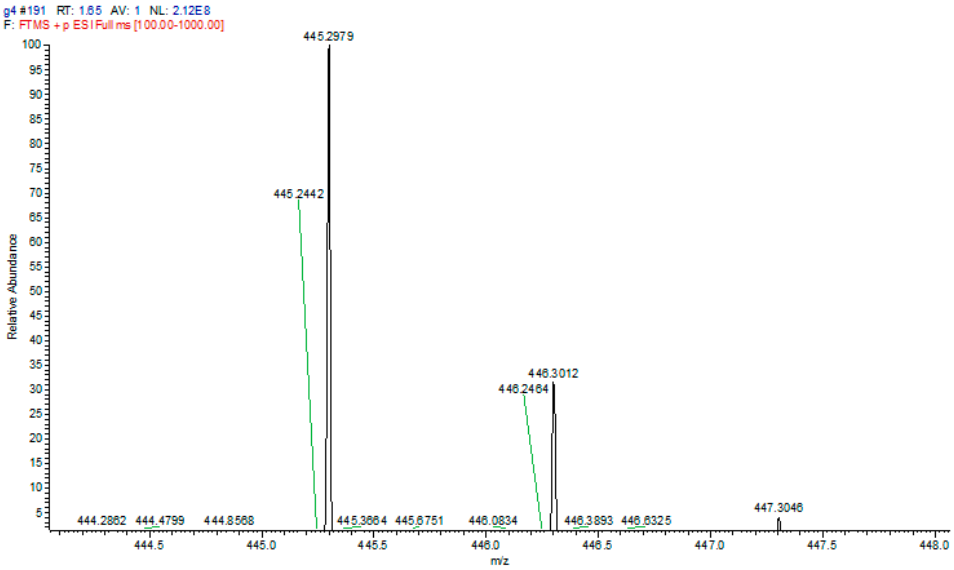

Idx	Formula	RDB	Delta mmu
1	$C_{28}H_{37}ON_4$	12.5	1.722

Caculated 445.2962 Found 445.2979

HRMS spectra of **6a4**

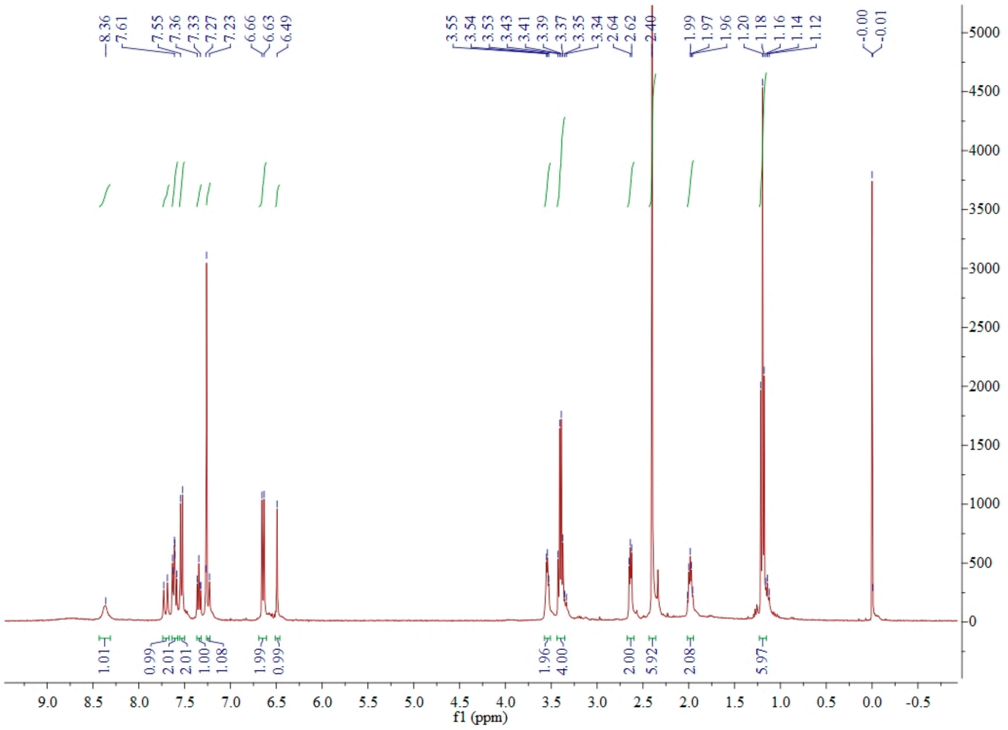

^{1}H NMR spectrum of **6b$_1$**

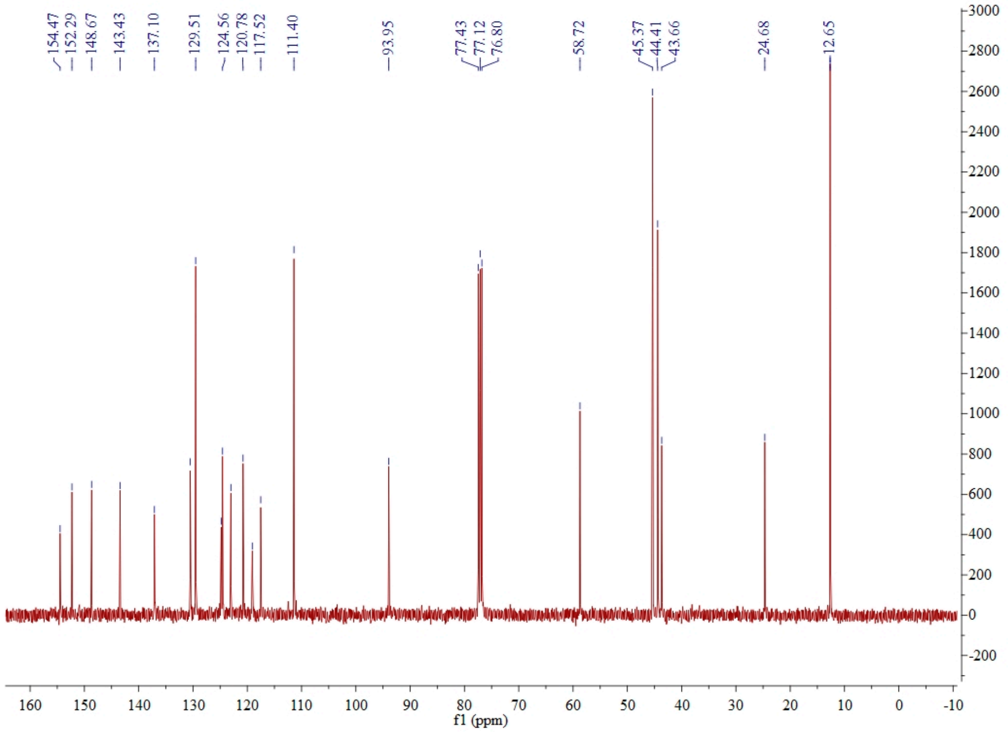

^{13}C NMR spectrum of **6b$_1$**

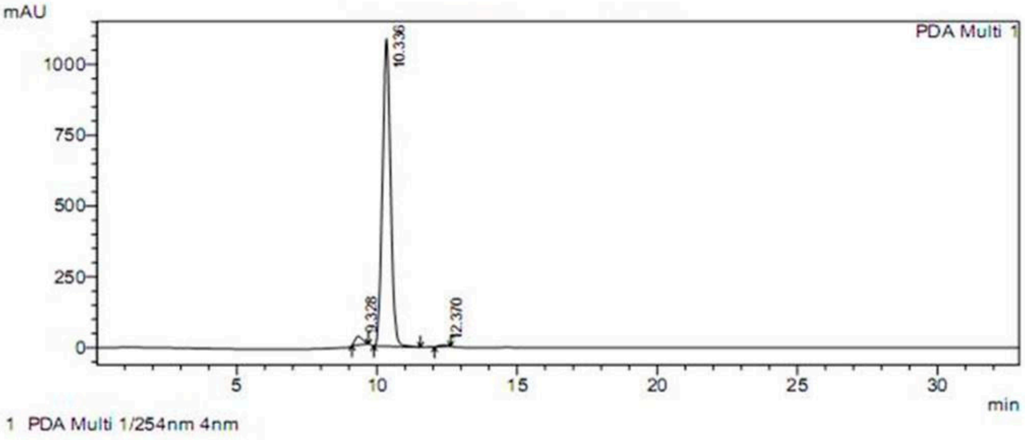

PeakTable

PDA Ch1 254nm 4nm

Peak#	Ret. Time	Area	Height	Area %	Height %
1	9.328	576203	30188	2.579	2.694
2	10.336	21664887	1085285	96.978	96.849
3	12.370	98901	5126	0.443	0.457
Total		22339991	1120599	100.000	100.000

HPLC spectrum of **6b1**

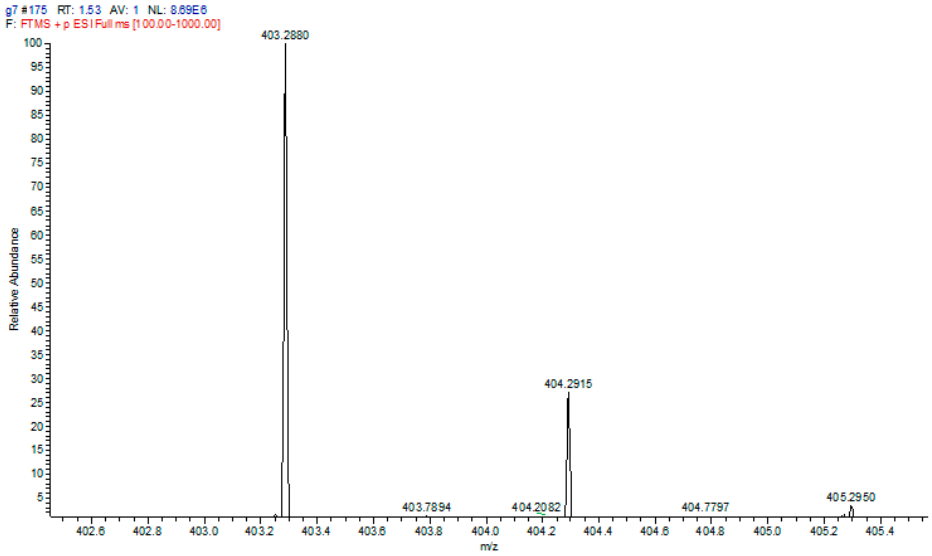

Idx	Formula	RDB	Delta mmu
1	$C_{26}H_{35}N_4$	11.5	2.336

Caculated 403.2856 Found 403.2880

HRMS spectra of **6b1**

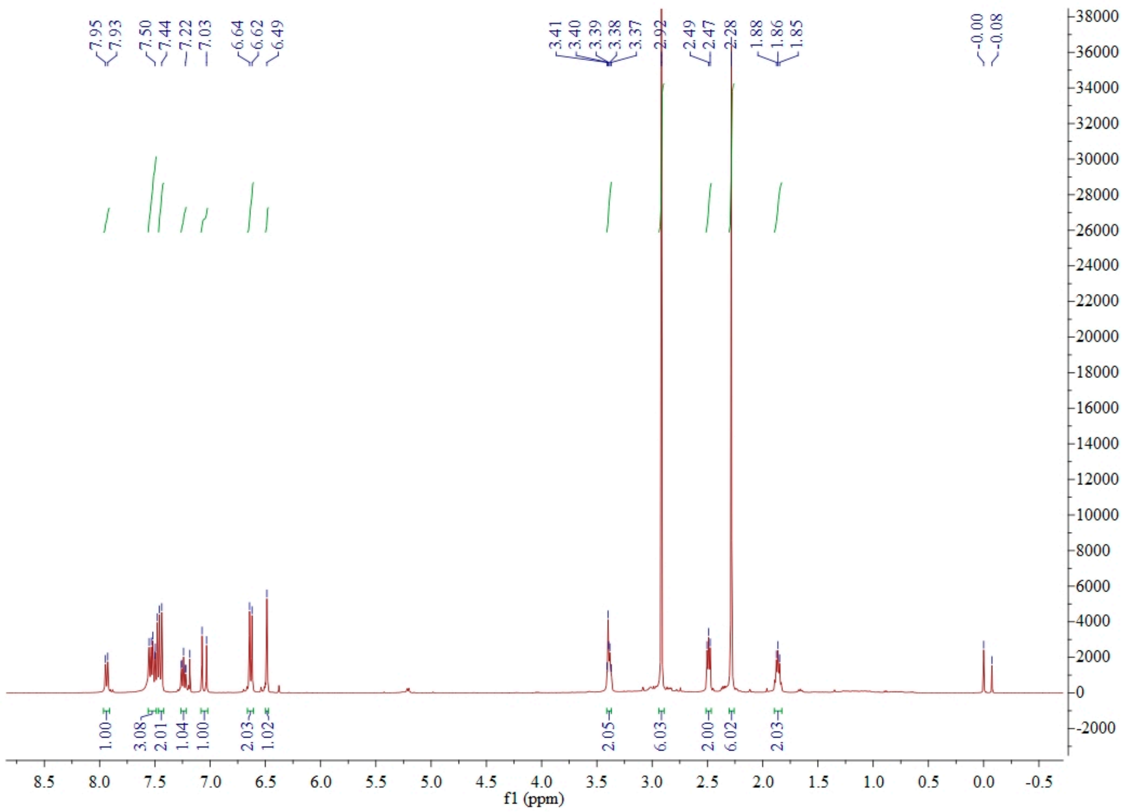

^{1}H NMR spectrum of **6b$_2$**

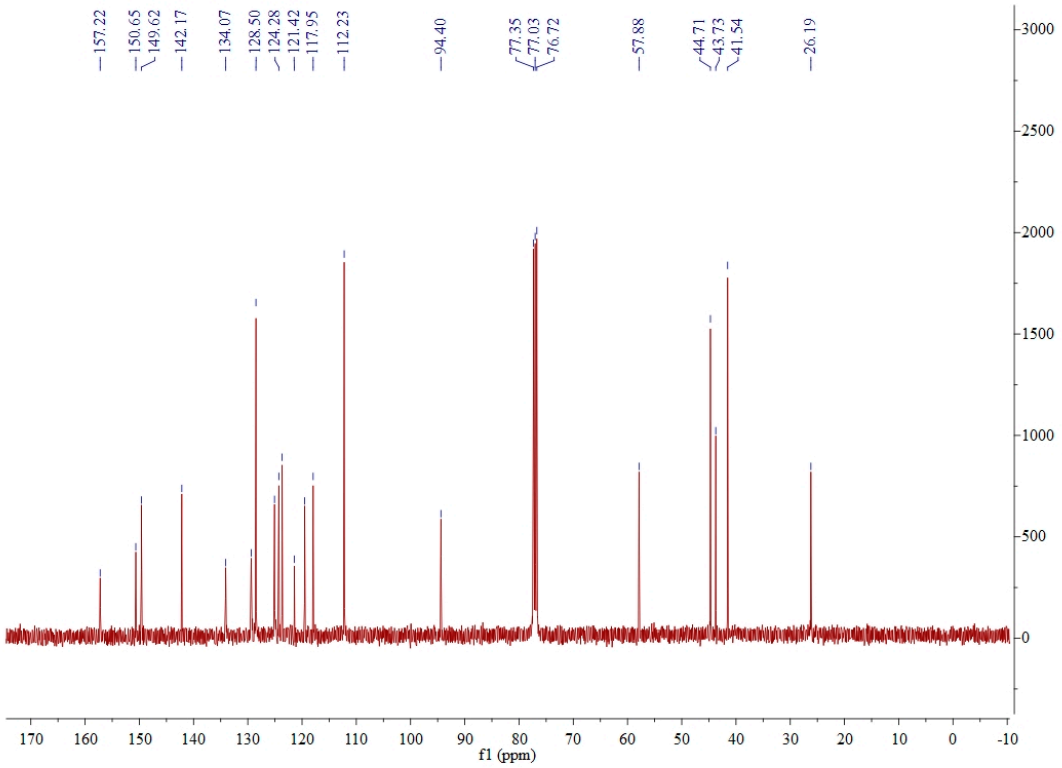

^{13}C NMR spectrum of **6b$_2$**

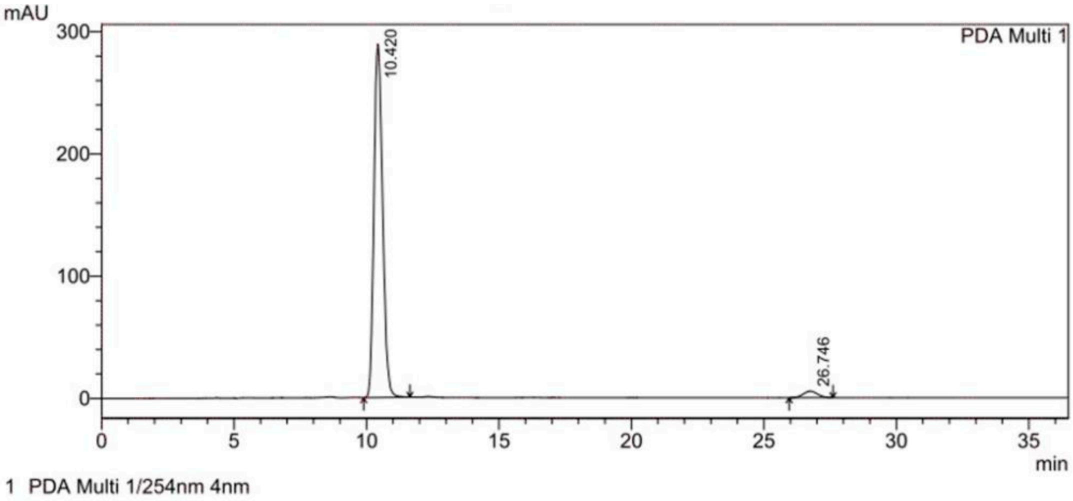

PeakTable

PDA Ch1 254nm 4nm

Peak#	Ret. Time	Area	Height	Area %	Height %
1	10.420	6566769	289073	97.022	98.189
2	26.746	201554	5333	2.978	1.811
Total		6768323	294406	100.000	100.000

HPLC spectrum of **6b**$_2$

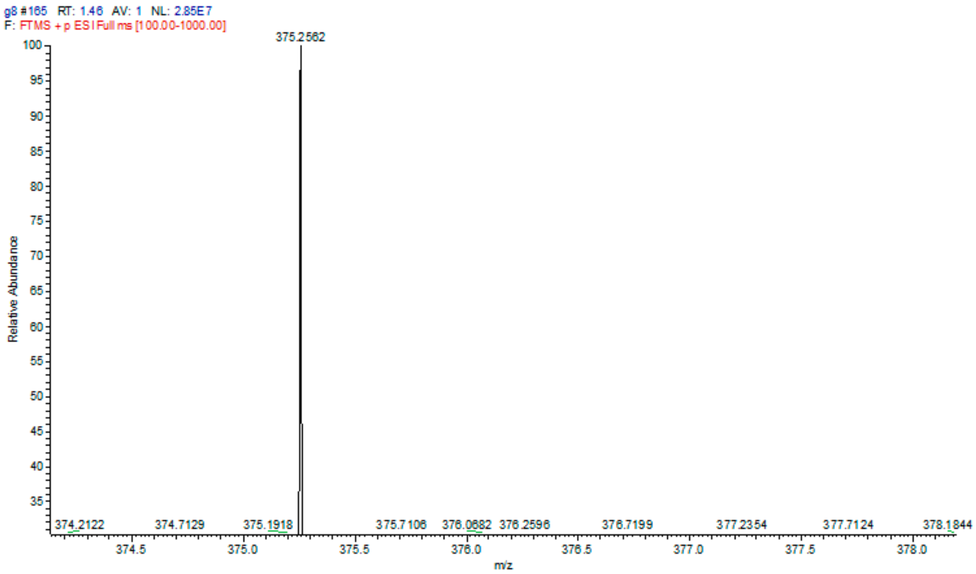

Idx	Formula	RDB	Delta mmu
1	$C_{24}H_{31}N_4$	11.5	1.876

Caculated 375.2543 Found 375.2562

HRMS spectra of **6b**$_2$

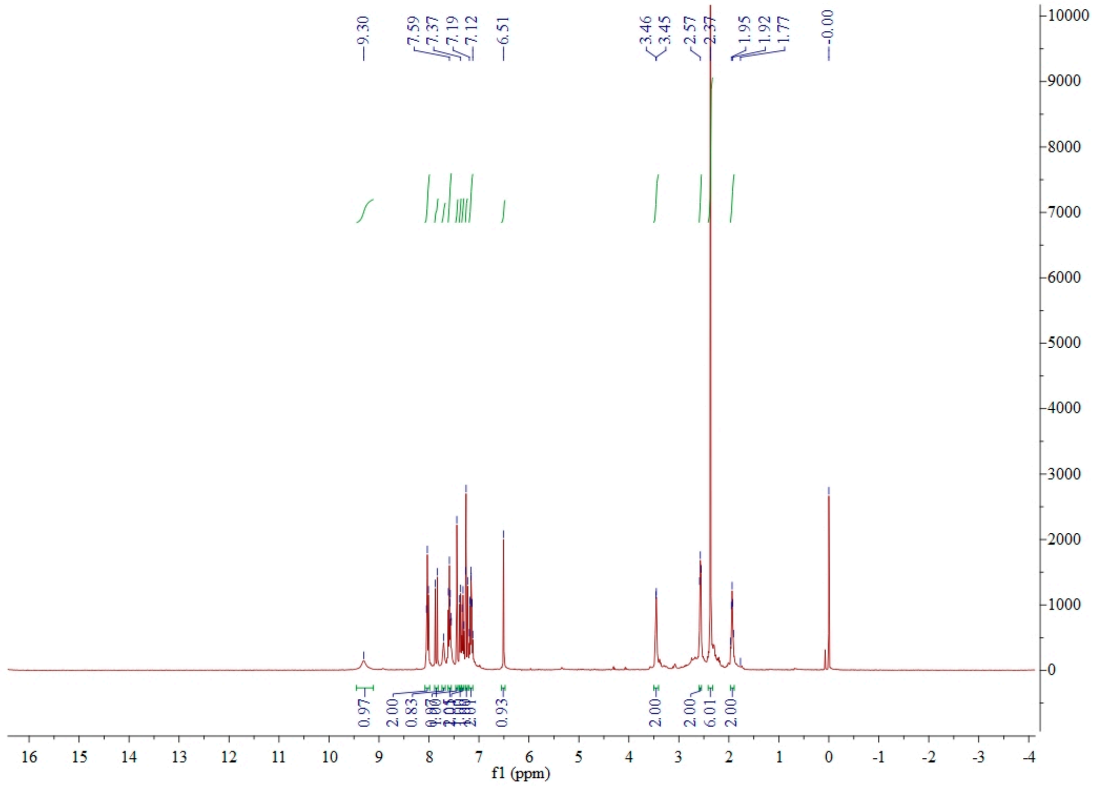

^{1}H NMR spectrum of **6b$_3$**

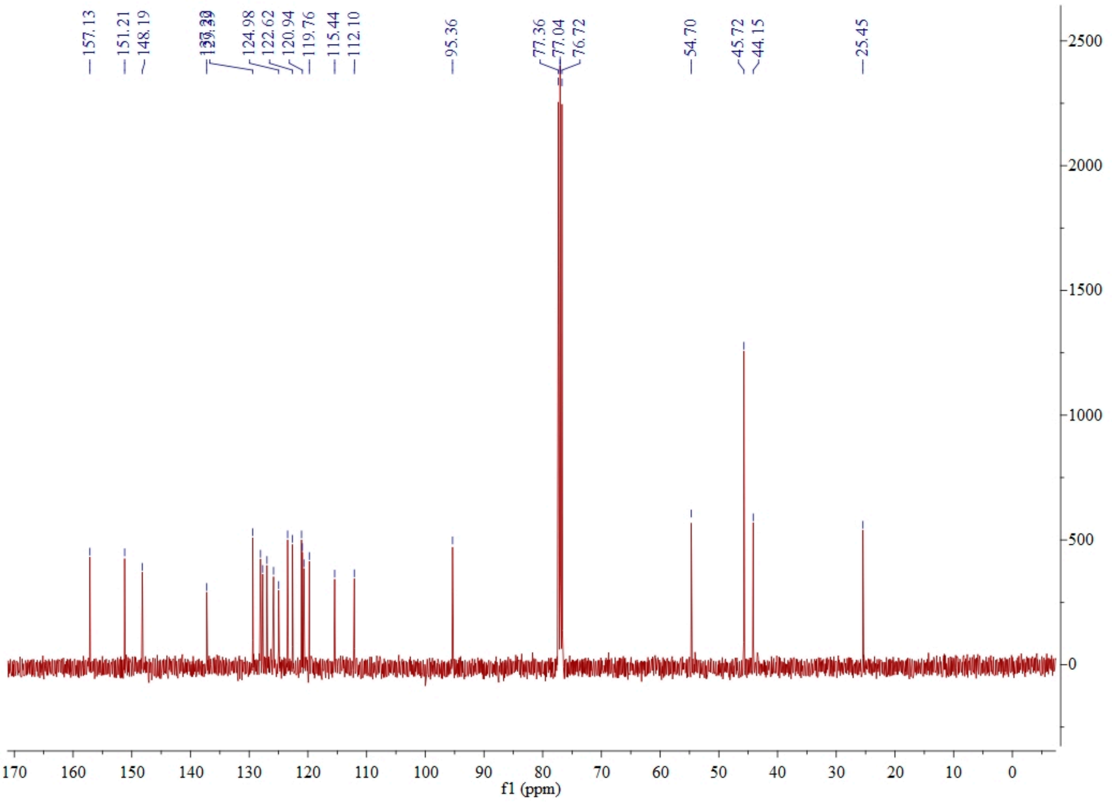

^{13}C NMR spectrum of **6b$_3$**

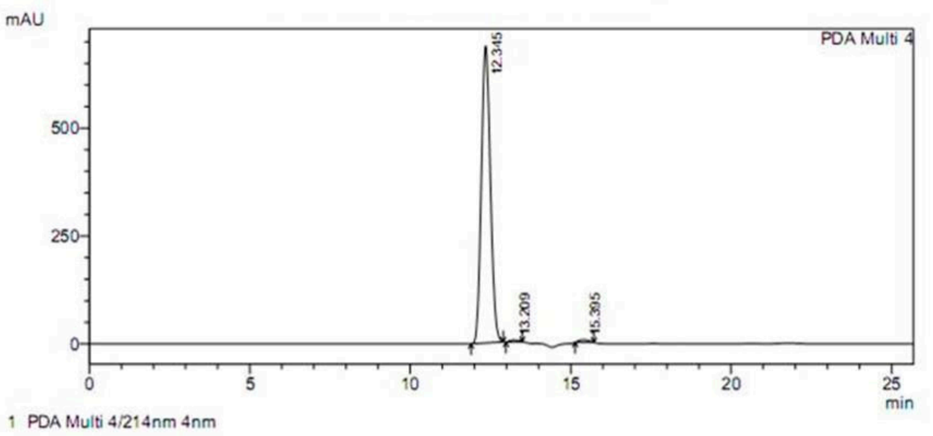

PeakTable

PDA Ch4 214nm 4nm

Peak#	Ret. Time	Area	Height	Area %	Height %
1	12.345	13376667	688830	98.453	98.509
2	13.209	77664	4391	0.572	0.628
3	15.395	132490	6032	0.975	0.863
Total		13586821	699253	100.000	100.000

HPLC spectrum of **6b3**

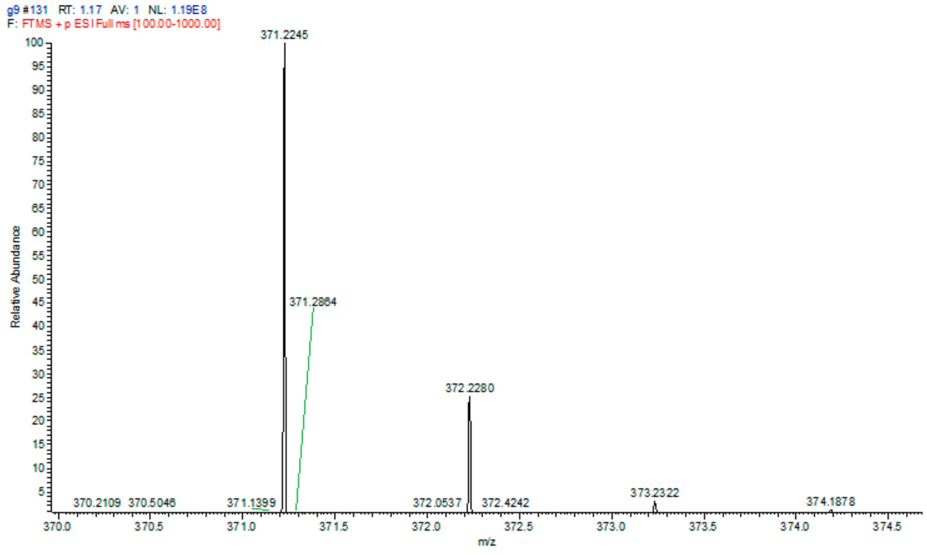

Idx	Formula	RDB	Delta mmu
1	$C_{24}H_{27}N_4$	13.5	1.497

Caculated 371.2230 Found 371.2245

HRMS spectra of **6b3**

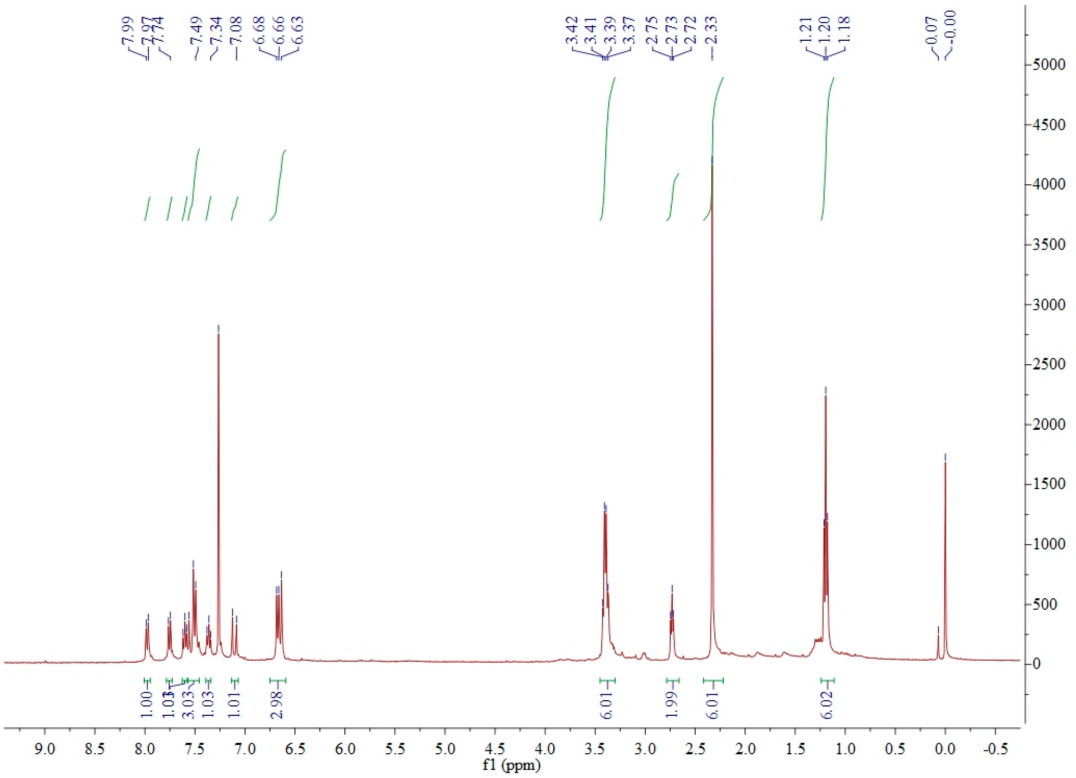

^{1}H NMR spectrum of **6c1**

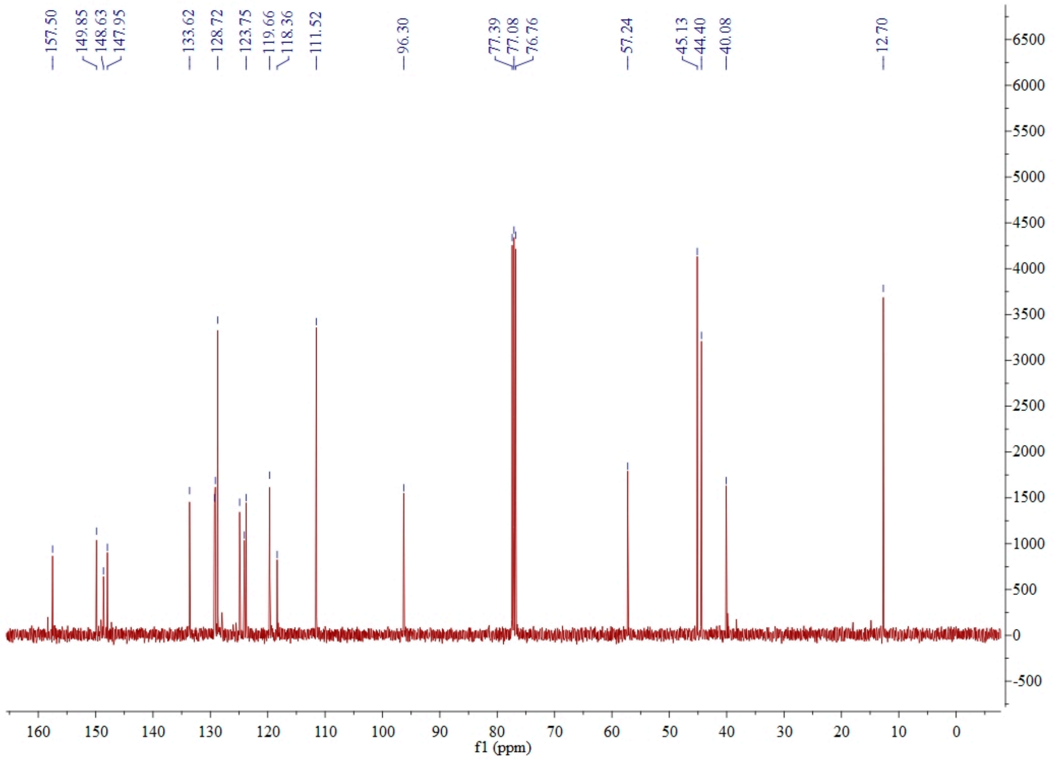

^{13}C NMR spectrum of **6c1**

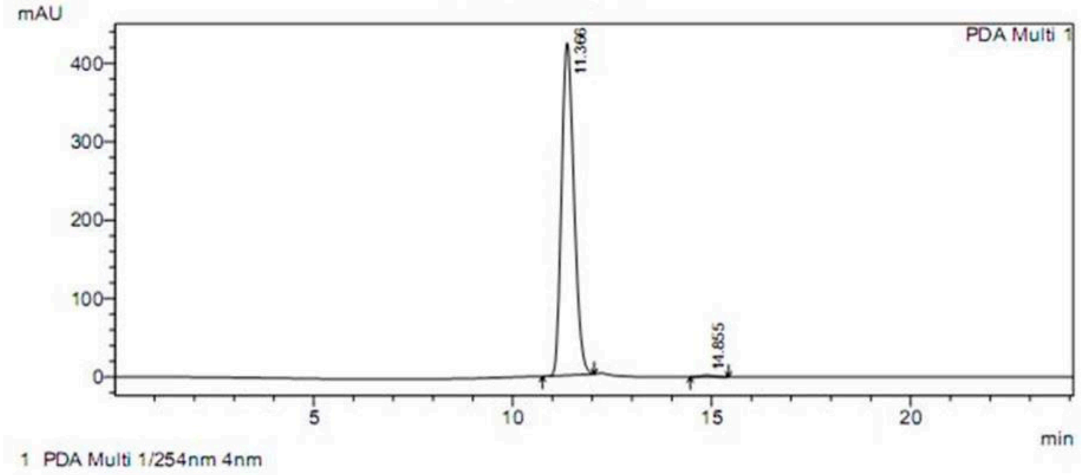

PeakTable

PDA Ch1 254nm 4nm

Peak#	Ret. Time	Area	Height	Area %	Height %
1	11.366	9198324	424318	99.377	99.425
2	14.855	57653	2454	0.623	0.575
Total		9255977	426772	100.000	100.000

HPLC spectrum of **6c1**

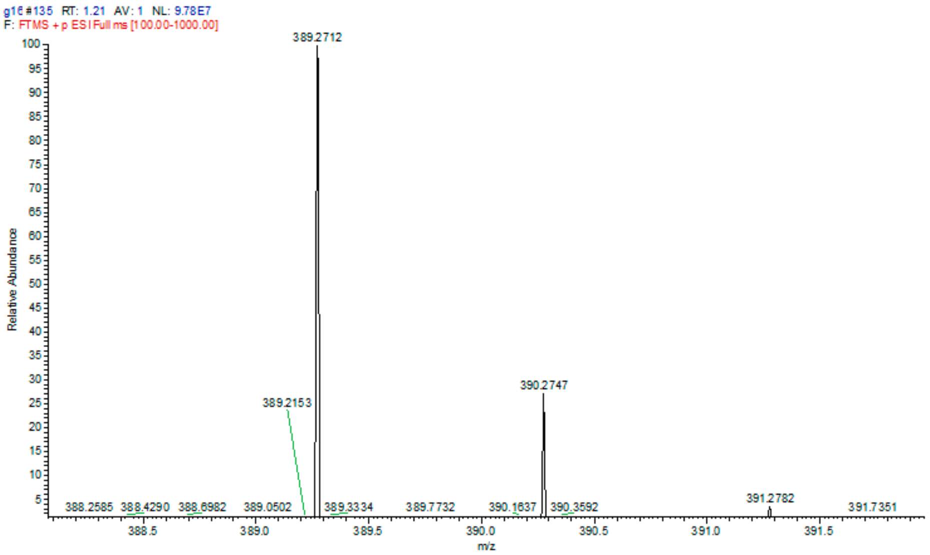

Idx	Formula	RDB	Delta mmu
1	$C_{25}H_{33}N_4$	11.5	1.266

Caculated 389.2700 Found 389.2712

HRMS spectra of **6c1**

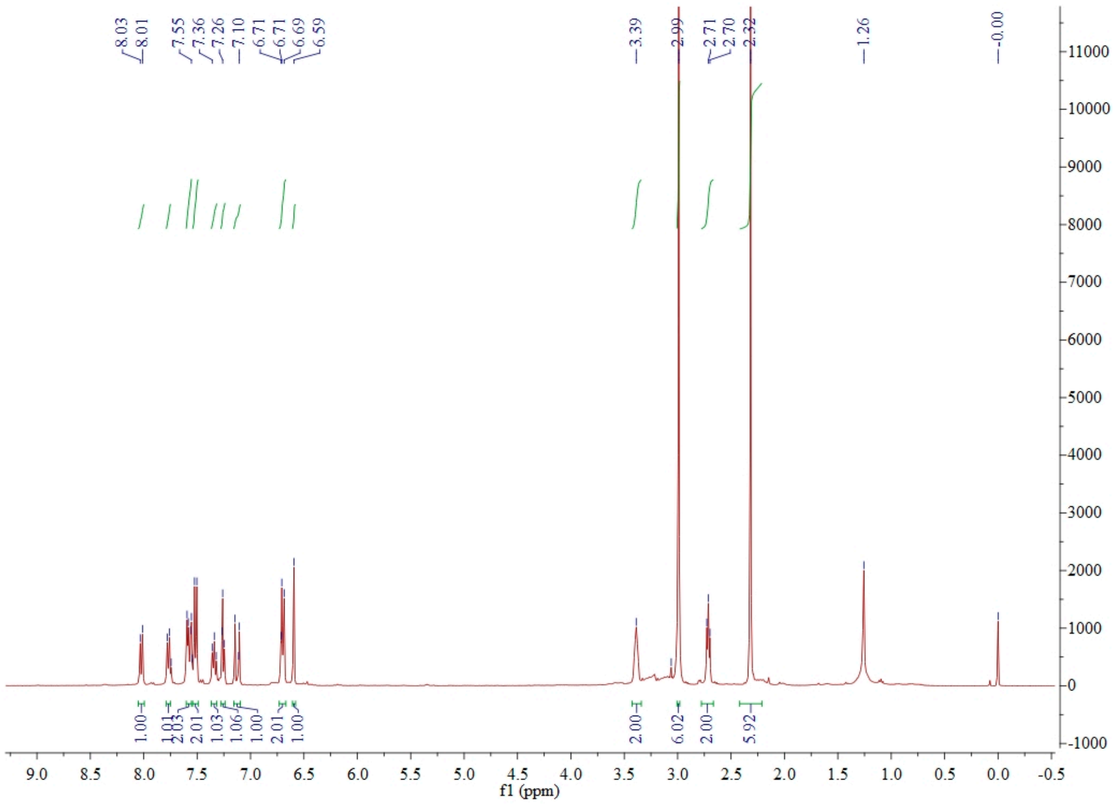

^{1}H NMR spectrum of **6c2**

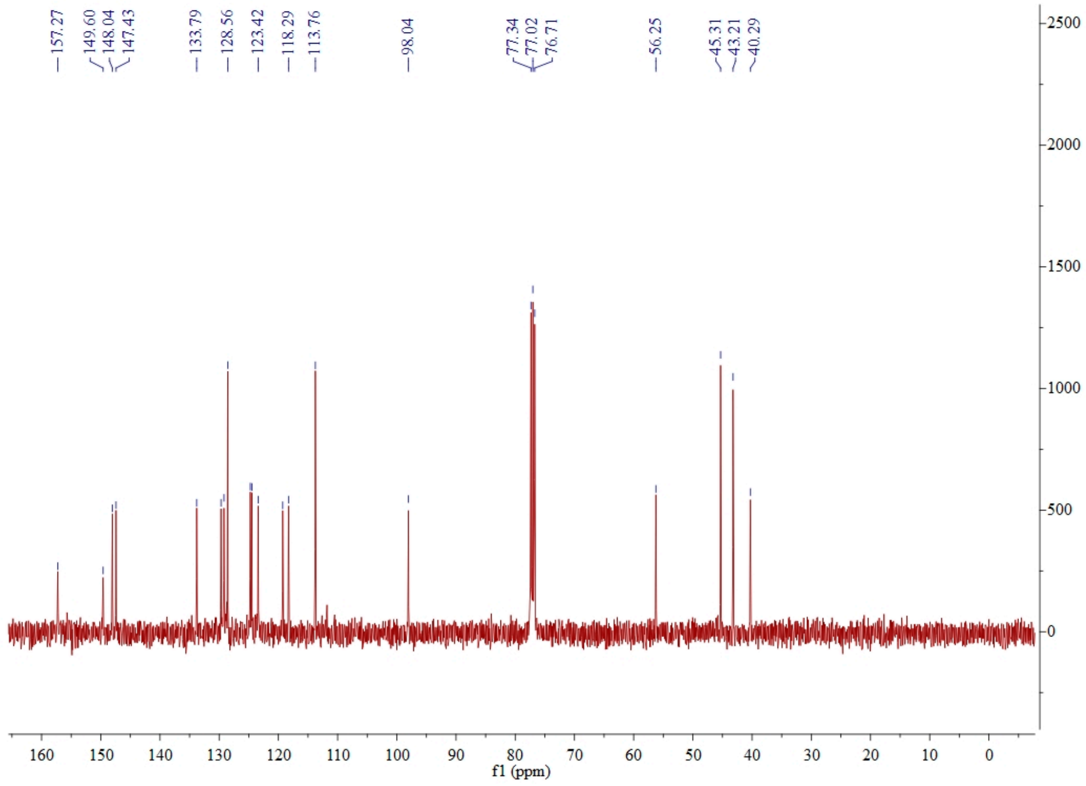

^{13}C NMR spectrum of **6c2**

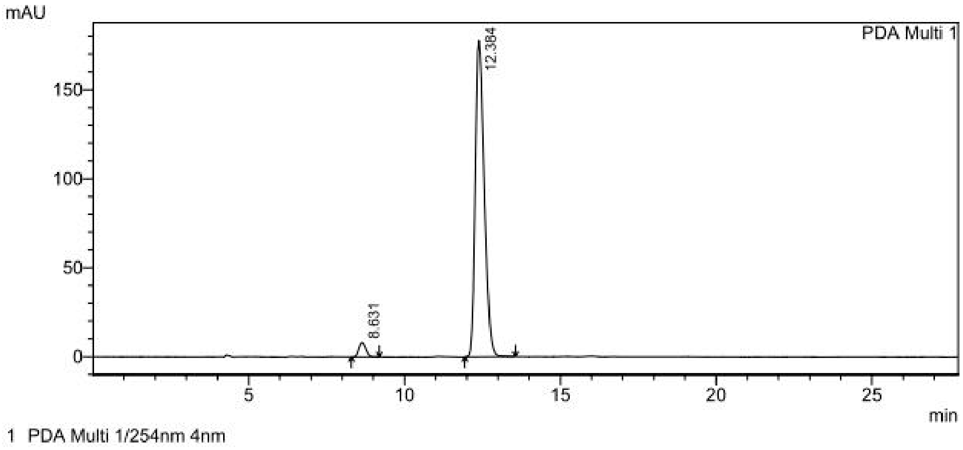

PeakTable

PDA Ch1 254nm 4nm

Peak#	Ret. Time	Area	Height	Area %	Height %
1	8.631	126388	7876	3.386	4.244
2	12.384	3606432	177683	96.614	95.756
Total		3732820	185559	100.000	100.000

HPLC spectrum of **6c2**

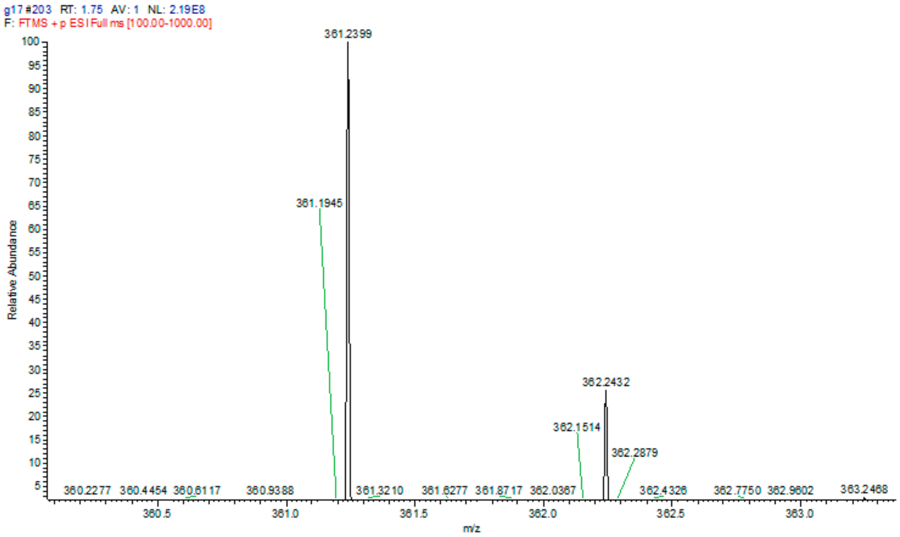

Idx	Formula	RDB	Delta mmu
1	$C_{23}H_{29}N_4$	11.5	1.227

Caculated 361.2387 Found 361.2399

HRMS spectra of **6c2**

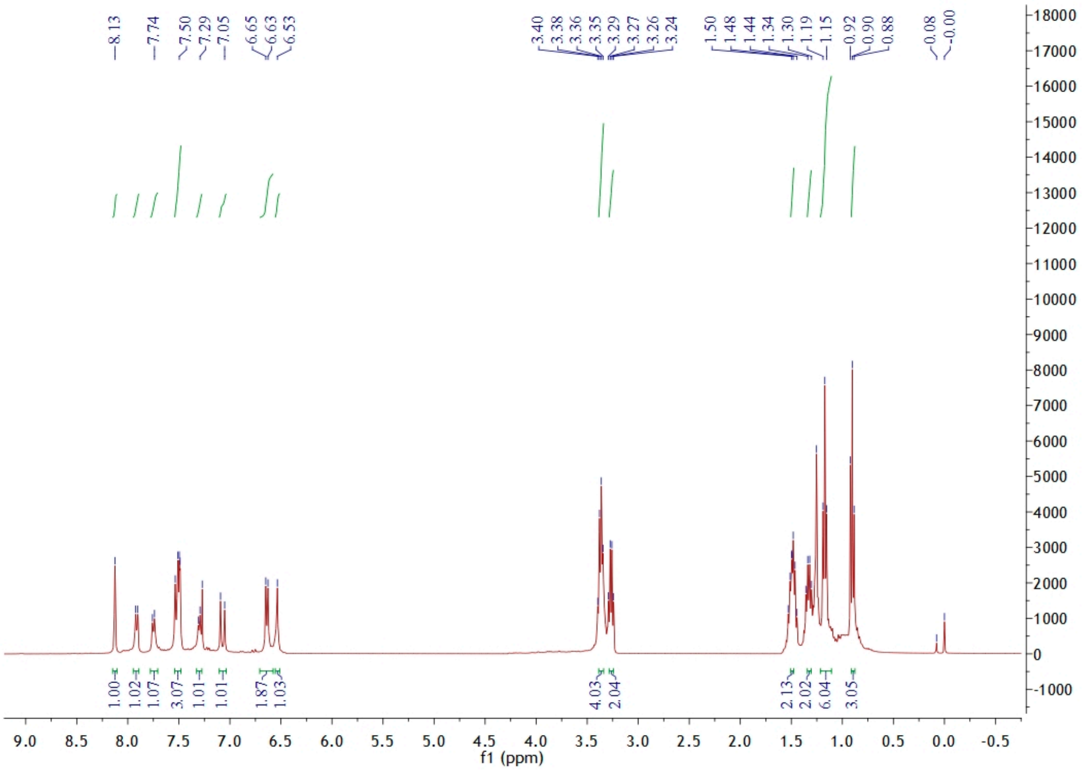

^{1}H NMR spectrum of **6d₁**

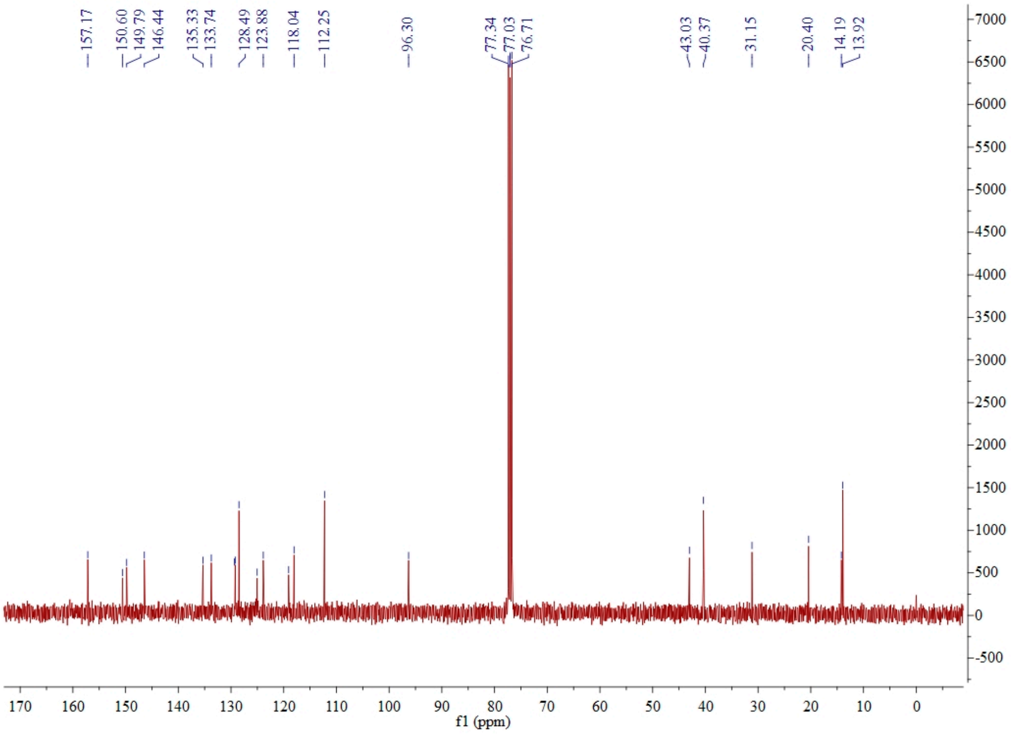

^{13}C NMR spectrum of **6d₁**

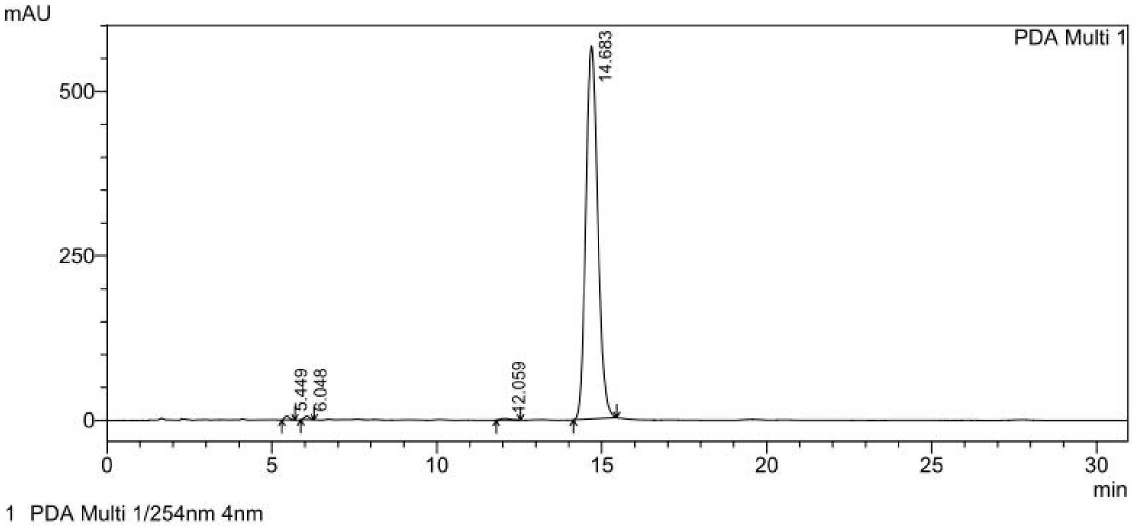

PeakTable

PDA Ch1 254nm 4nm

Peak#	Ret. Time	Area	Height	Area %	Height %
1	5.449	58406	6111	0.408	1.053
2	6.048	63630	5403	0.444	0.931
3	12.059	49454	2383	0.345	0.411
4	14.683	14143754	566399	98.802	97.605
Total		14315244	580296	100.000	100.000

HPLC spectrum of **6d$_1$**

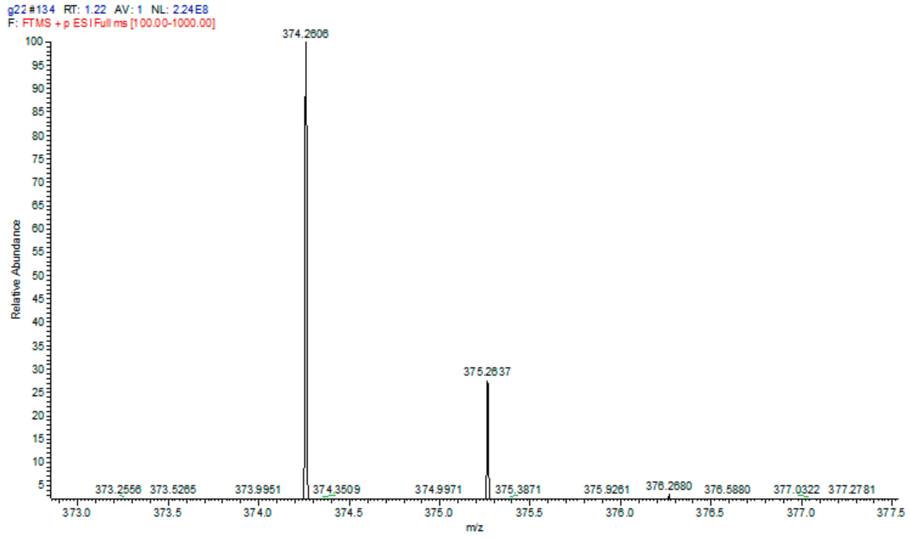

Idx	Formula	RDB	Delta mmu
1	$C_{25}H_{32}N_3$	11.5	1.485

Caculated 374.2591 Found 374.2606

HRMS spectra of **6d$_1$**

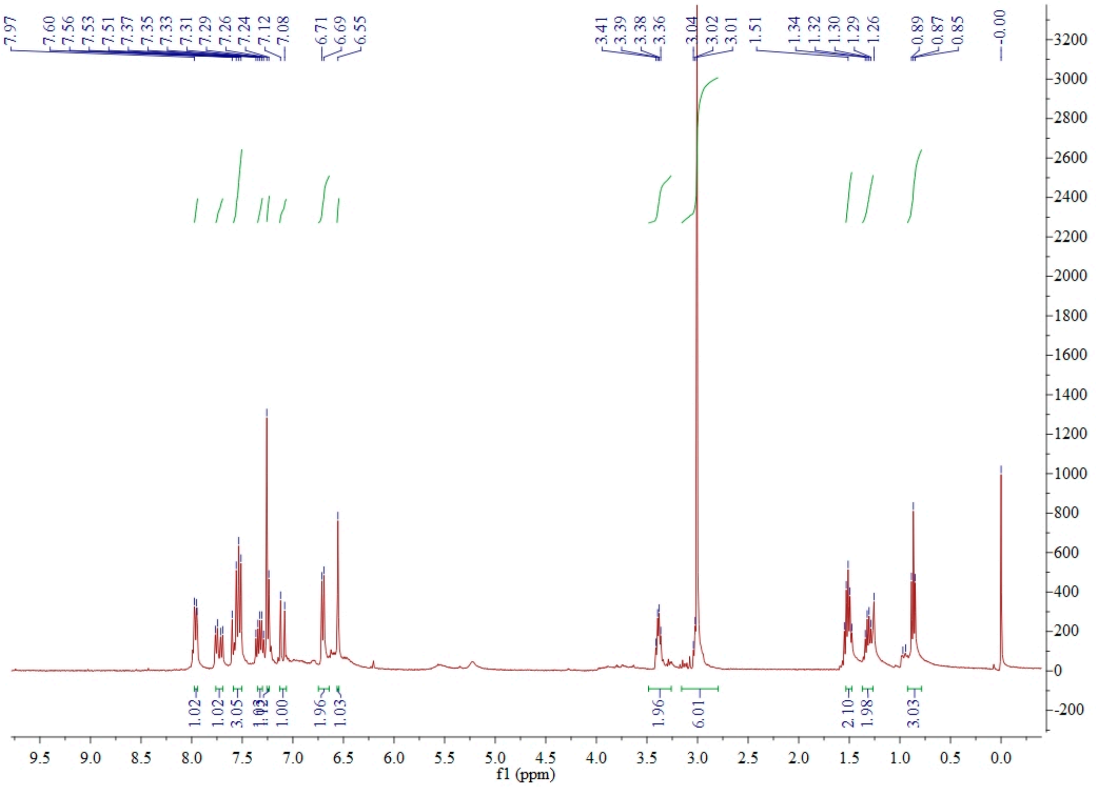

¹H NMR spectrum of **6d₂**

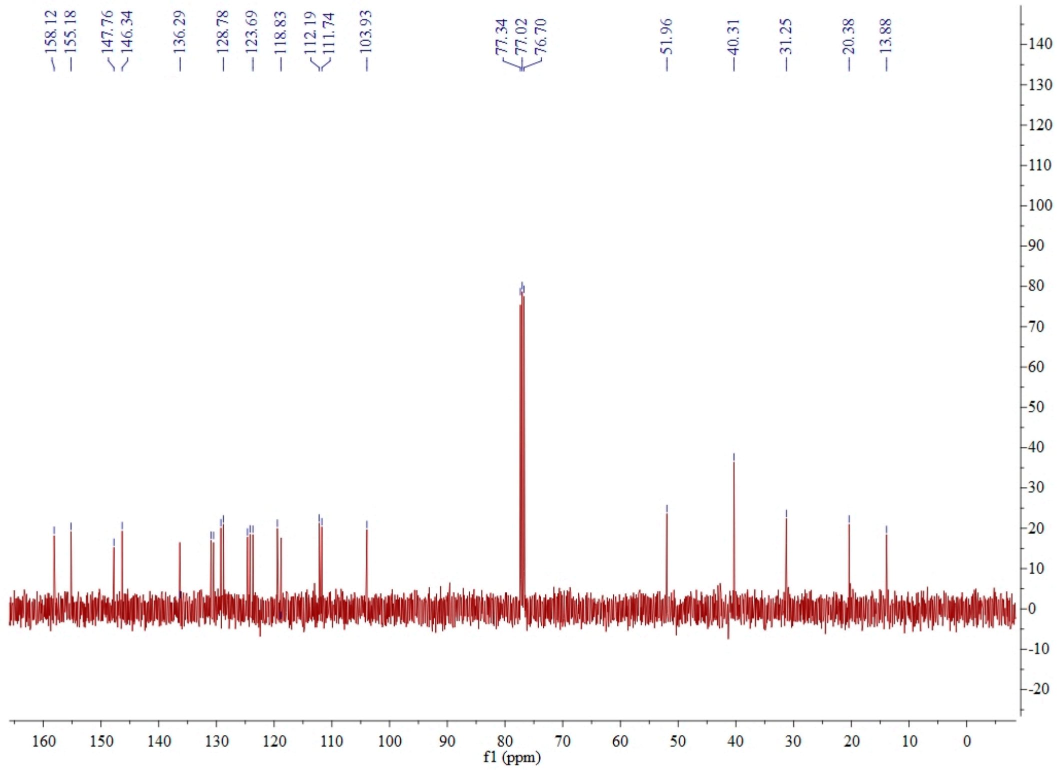

¹³C NMR spectrum of **6d₂**

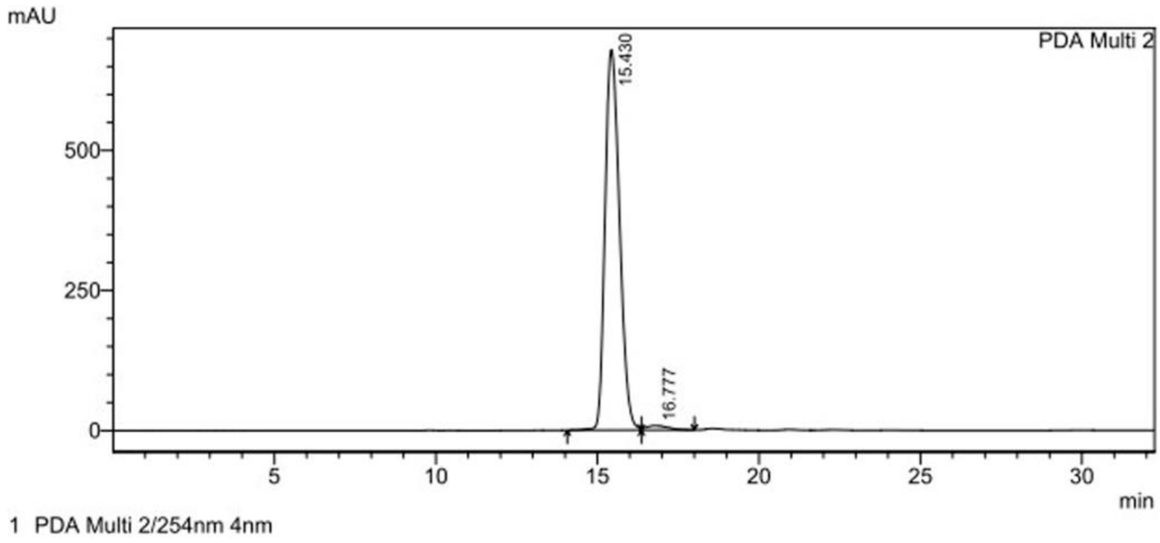

PeakTable

PDA Ch2 254nm 4nm

Peak#	Ret. Time	Area	Height	Area %	Height %
1	15.430	21153180	679537	98.265	98.866
2	16.777	373403	7793	1.735	1.134
Total		21526583	687330	100.000	100.000

HPLC spectrum of **6d$_2$**

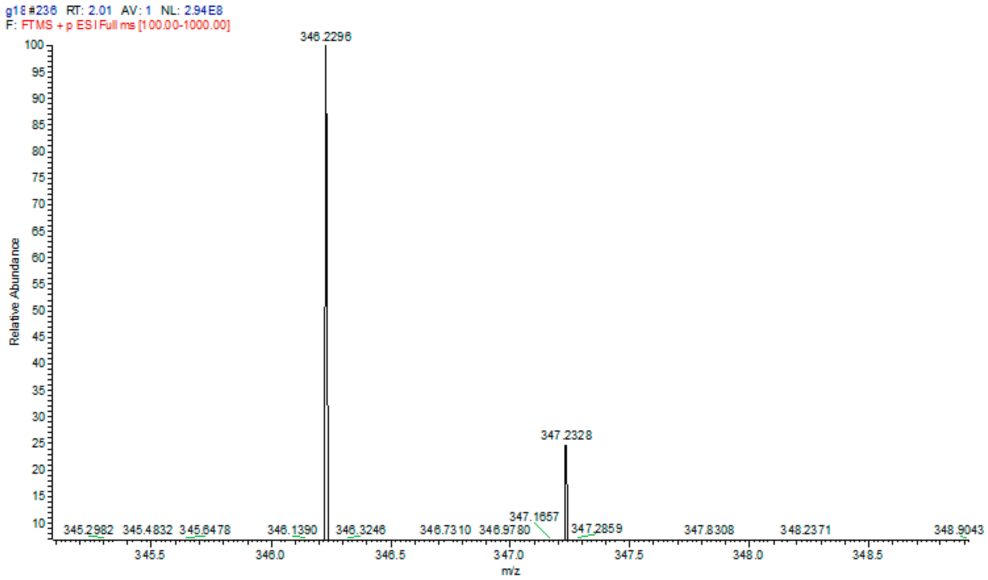

Idx	Formula	RDB	Delta mmu
1	$C_{23}H_{28}N_3$	11.5	1.806

Caculated 346.2278 Found 346.2296

HRMS spectra of **6d$_2$**

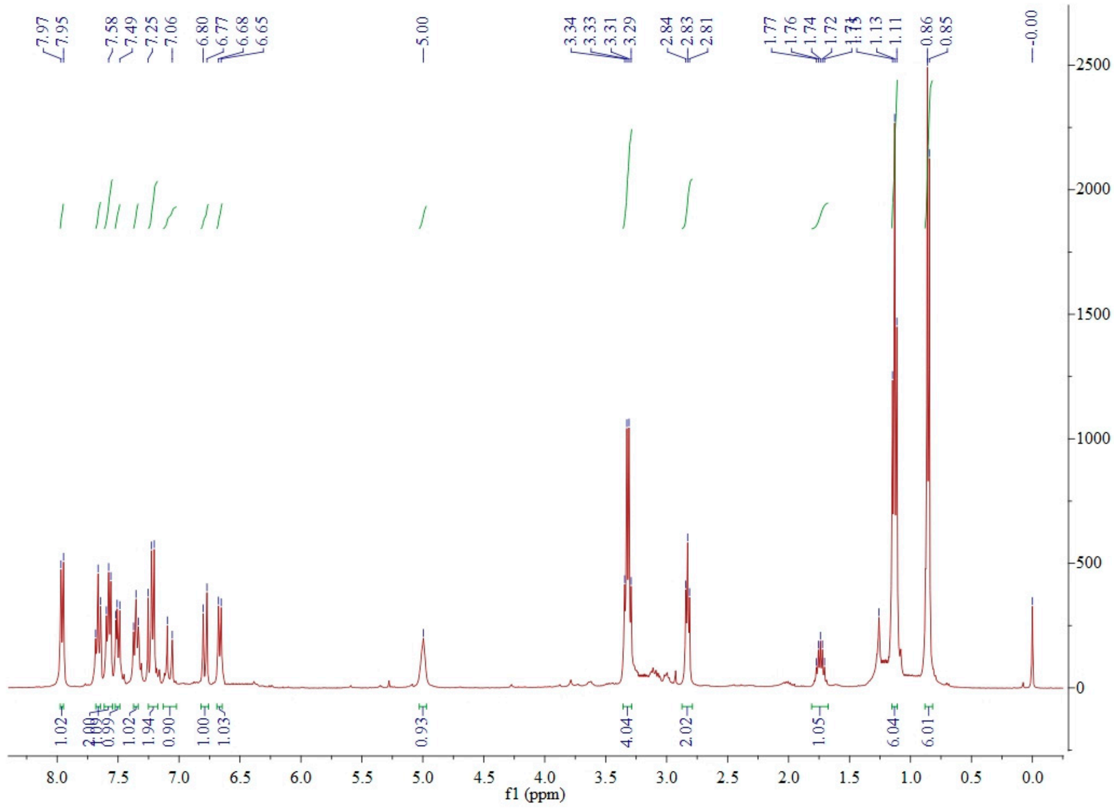

^{1}H NMR spectrum of **6e**$_1$

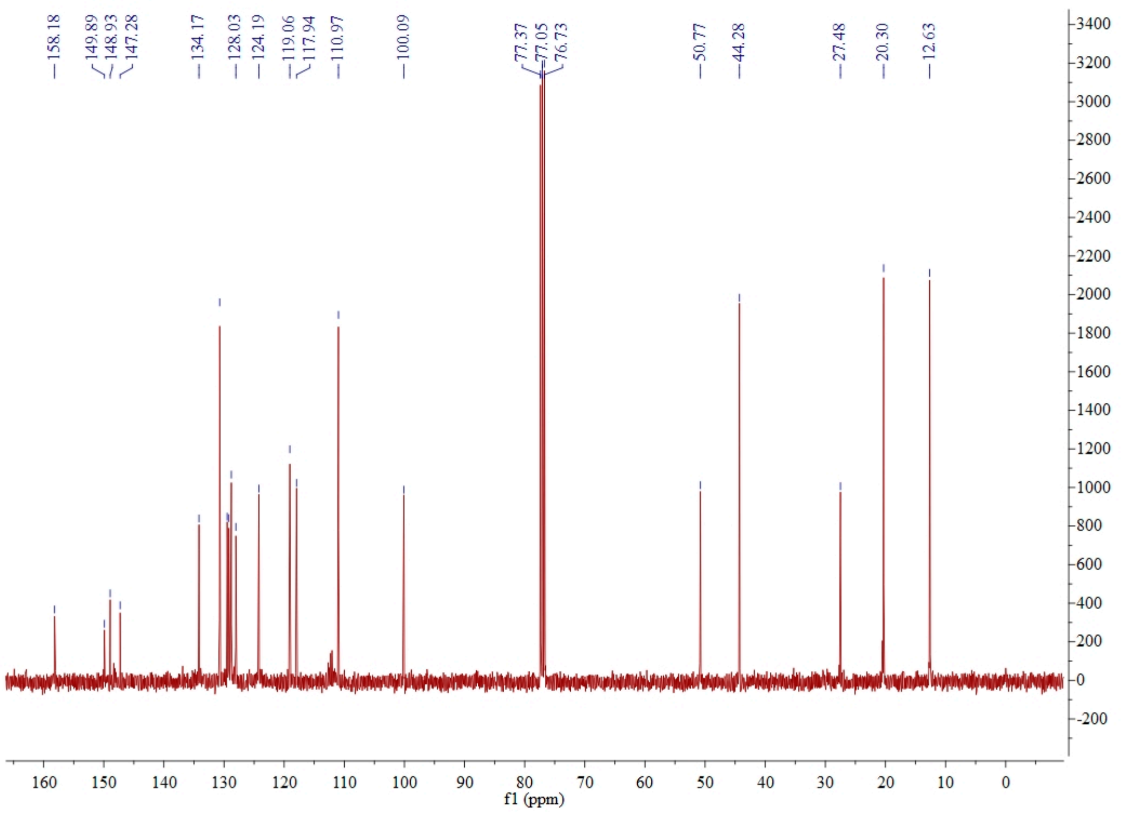

^{13}C NMR spectrum of **6e**$_1$

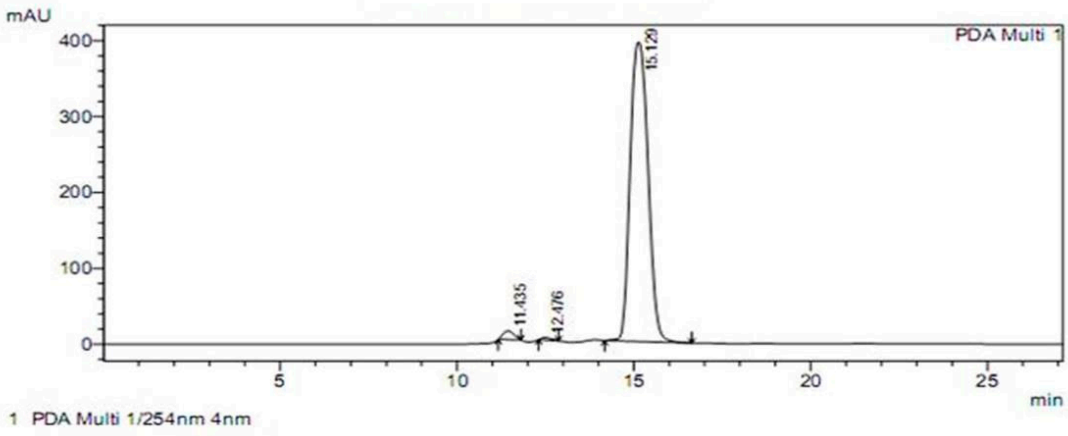

PeakTable

PDA Ch1 254nm 4nm

Peak#	Ret. Time	Area	Height	Area %	Height %
1	11.435	251598	11831	1.777	2.888
2	12.476	67460	3261	0.476	0.796
3	15.129	13840429	394620	97.747	96.317
Total		14159487	409712	100.000	100.000

HPLC spectrum of **6e1**

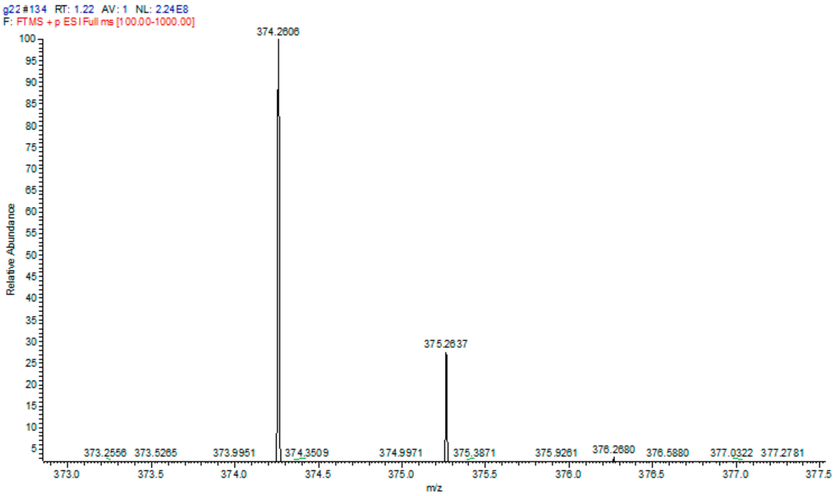

Idx	Formula	RDB	Delta mmu
1	$C_{25}H_{32}N_3$	11.5	1.485

Caculated 374.2591 Found 374.2606

HRMS spectra of **6e1**

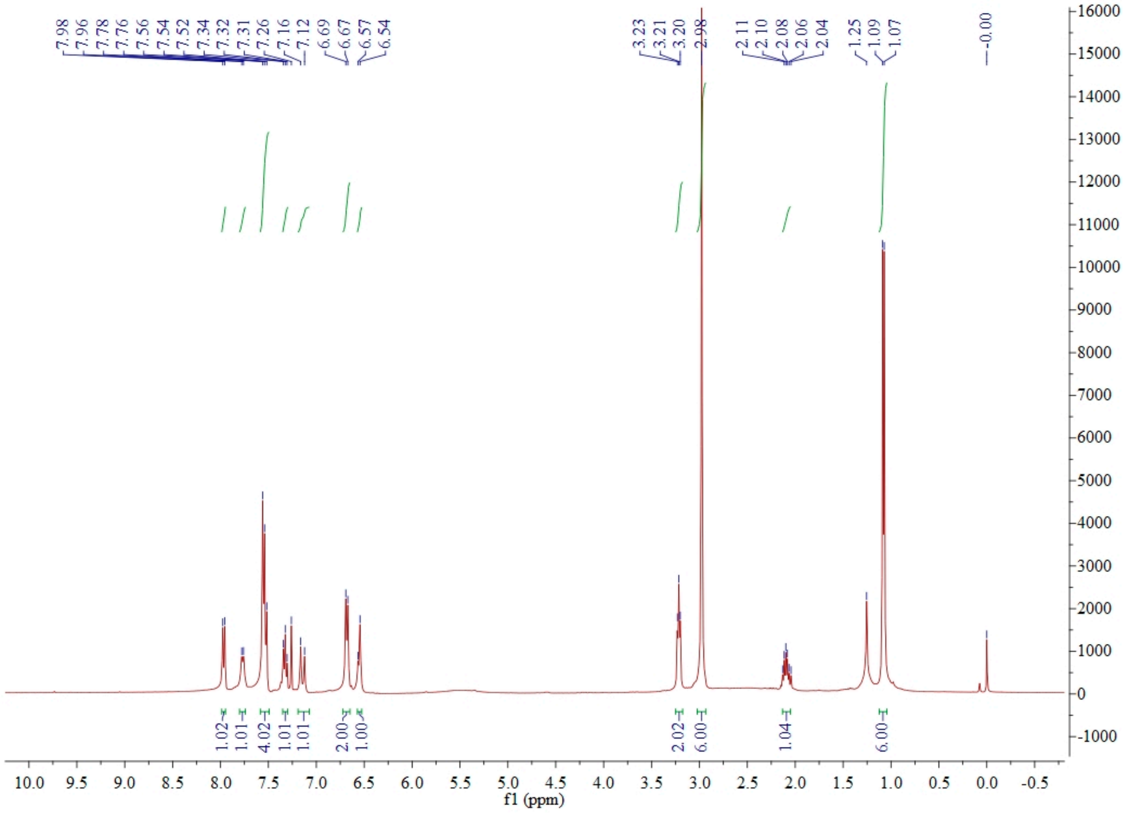

^{1}H NMR spectrum of **6e$_2$**

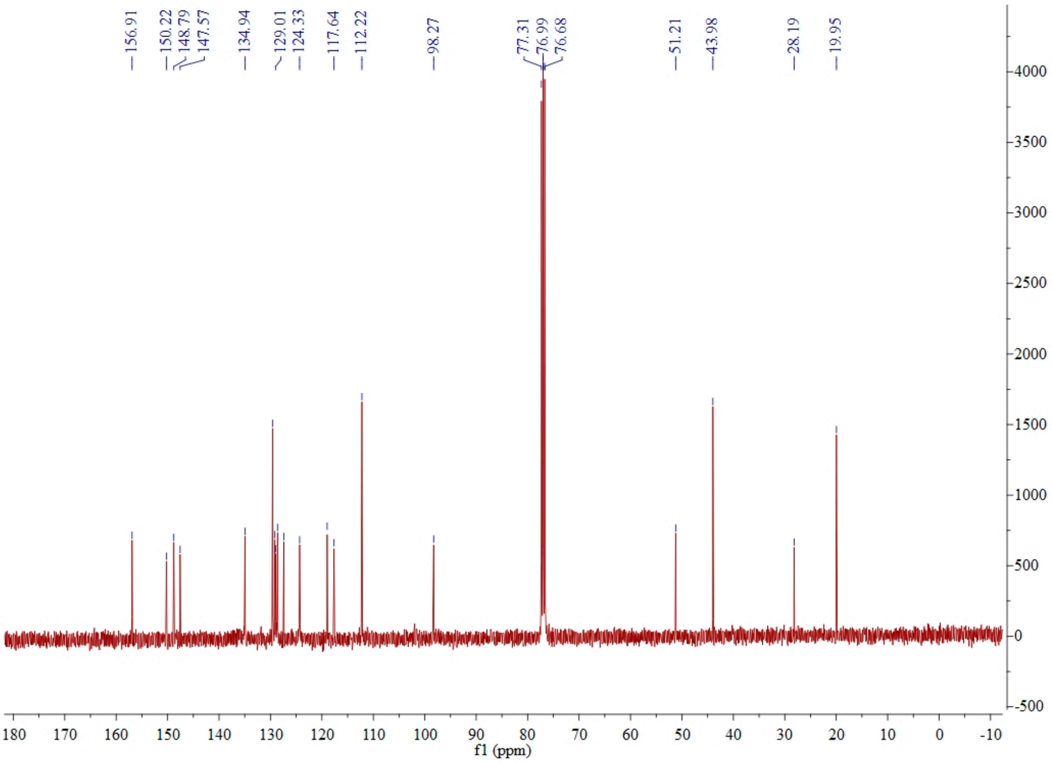

^{13}C NMR spectrum of **6e$_2$**

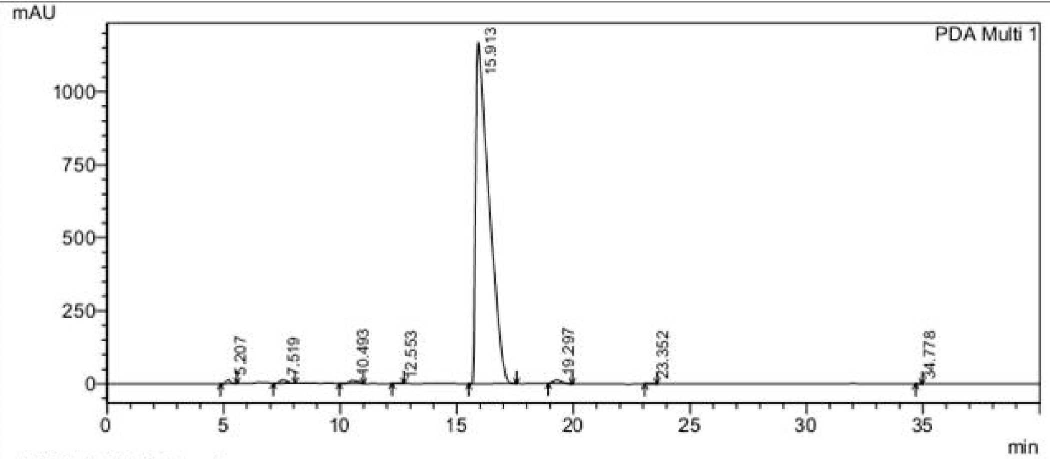

1 PDA Multi 1/254nm 4nm

PeakTable

PDA Ch1 254nm 4nm

Peak#	Ret. Time	Area	Height	Area %	Height %
1	5.207	169692	13183	0.357	1.085
2	7.519	256451	10424	0.540	0.858
3	10.493	273322	9732	0.575	0.801
4	12.553	10233	472	0.022	0.039
5	15.913	46522556	1169482	97.899	96.250
6	19.297	284292	11424	0.598	0.940
7	23.352	3565	213	0.008	0.018
8	34.778	1048	118	0.002	0.010
Total		47521158	1215049	100.000	100.000

HPLC spectrum of **6e2**

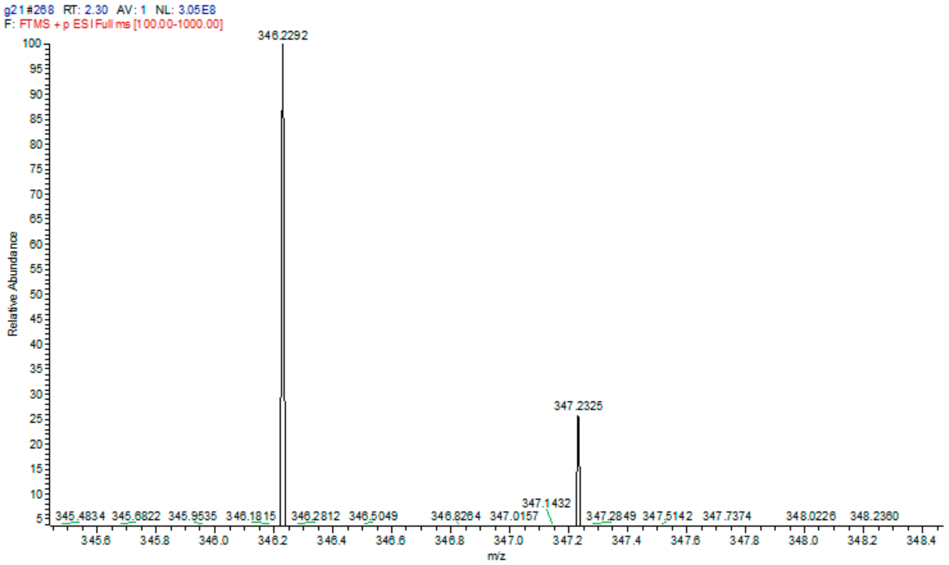

Idx	Formula	RDB	Delta mmu
1	$C_{23}H_{28}N_3$	11.5	1.476

Caculated 346.2278 Found 346.2292

HRMS spectra of **6e2**

Table S1. cytotoxicity (IC_{50}) on SH-SY5Y cells of prepared compounds.

Compd [a]	IC_{50} [μM][a]	Compd a	IC_{50} [μM][a]
**6a1**	189.2 ± 0.8	**6c2**	127.5 ± 0.4
**6a2**	187.5 ± 0.2	**6d1**	119.3 ± 0.7
**6a3**	147.8 ± 0.3	**6d2**	112.4 ± 0.6
**6a4**	145.2 ± 0.9	**6e1**	143.6 ± 0.7
**6b1**	253.7 ± 0.4	**6e2**	141.9 ± 0.5
**6b2**	228.1 ± 0.5	**6f1**	111.2 ± 0.4
**6b3**	168.1 ± 0.2	**6f2**	106.3 ± 0.4
**6c1**	154.3 ± 0.5		

a Values are the mean ± SEM of three independent measurements.

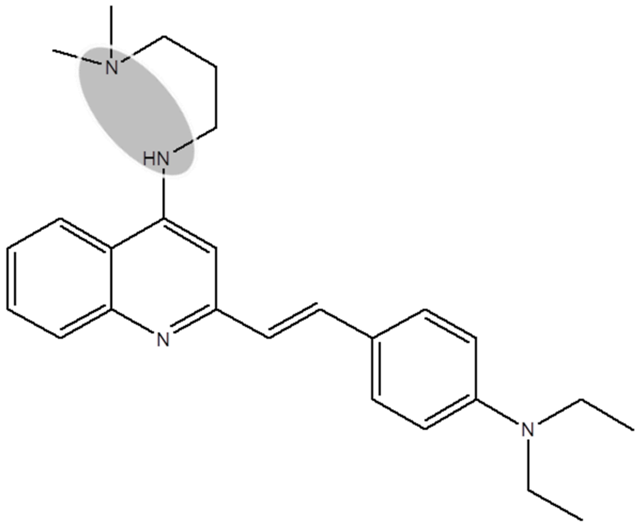

Scheme S1. The coordination model of compound **6b1** for Cu^{2+} (Chelating motif in gray shade).
